# SpaMask: Dual masking graph autoencoder with contrastive learning for spatial transcriptomics

**DOI:** 10.1371/journal.pcbi.1012881

**Published:** 2025-04-03

**Authors:** Wenwen Min, Donghai Fang, Jinyu Chen, Shihua Zhang

**Affiliations:** 1 School of Information Science and Engineering, Yunnan University, Kunming, Yunnan, China; 2 School of Mathematics, Statistics and Mechanics, Beijing University of Technology, Beijing, China; 3 NCMIS, CEMS, RCSDS, Academy of Mathematics and Systems Science, Chinese Academy of Sciences, Beijing, China; 4 School of Mathematical Sciences, University of Chinese Academy of Sciences, Beijing, China; 5 Key Laboratory of Systems Health Science of Zhejiang Province, School of Life Science, Hangzhou Institute for Advanced Study, University of Chinese Academy of Sciences, Hangzhou, Zhejiang, China; University of Pittsburgh, UNITED STATES OF AMERICA

## Abstract

Understanding the spatial locations of cell within tissues is crucial for unraveling the organization of cellular diversity. Recent advancements in spatial resolved transcriptomics (SRT) have enabled the analysis of gene expression while preserving the spatial context within tissues. Spatial domain characterization is a critical first step in SRT data analysis, providing the foundation for subsequent analyses and insights into biological implications. Graph neural networks (GNNs) have emerged as a common tool for addressing this challenge due to the structural nature of SRT data. However, current graph-based deep learning approaches often overlook the instability caused by the high sparsity of SRT data. Masking mechanisms, as an effective self-supervised learning strategy, can enhance the robustness of these models. To this end, we propose SpaMask, dual masking graph autoencoder with contrastive learning for SRT analysis. Unlike previous GNNs, SpaMask masks a portion of spot nodes and spot-to-spot edges to enhance its performance and robustness. SpaMask combines Masked Graph Autoencoders (MGAE) and Masked Graph Contrastive Learning (MGCL) modules, with MGAE using node masking to leverage spatial neighbors for improved clustering accuracy, while MGCL applies edge masking to create a contrastive loss framework that tightens embeddings of adjacent nodes based on spatial proximity and feature similarity. We conducted a comprehensive evaluation of SpaMask on eight datasets from five different platforms. Compared to existing methods, SpaMask achieves superior clustering accuracy and effective batch correction.

## Author summary

Spatial resolved transcriptomics (SRT) is a powerful technology for mapping gene expression within tissue architectures, but accurately analyzing and interpreting these complex data sets remains a challenge. In this study, we introduce SpaMask, a novel approach that combines Masked Graph Autoencoders (MGAE) and Masked Graph Contrastive Learning (MGCL) to improve clustering accuracy and handle batch effects in SRT data. By leveraging a shared graph encoder between MGAE and MGCL modules, SpaMask effectively captures the relationship between gene expression and spatial coordinates, leading to more precise identification of spatial domains. A detailed evaluation of SpaMask was performed on eight datasets sourced from five separate platforms. The results demonstrate that SpaMask outperforms existing methods in terms of clustering accuracy and robustness. This work has important implications for advancing spatial transcriptomics, providing a more accurate framework for understanding tissue organization and cellular diversity in health and disease.

## 1. Introduction

In complex biological organisms, cells are organized into similar clusters within their spatial environments [1,2]. This intricate tissue arrangement reflects dynamic interactions among cells and their specialized functions [3]. Recent advancements in spatially resolved transcriptomics (SRT) technologies—such as ST with a resolution of 100 µm, 10x Visium [4] at 55 µm, and Stereo-seq [5] at 220 nm—enable comprehensive genomic analyses at the multicellular and even single-cell levels, capturing gene expression corresponding to specific spatial locations, referred to as spots [6,7]. This spatial information enhances researchers’ understanding of various biological processes influencing diseases, providing a solid foundation for future investigations [8].

A significant computational task in contemporary SRT data analysis is the identification of regions with similar spatial expression patterns, termed spatial domains [6,9,10]. This process primarily involves both non-spatial and spatial clustering methods [7]. Traditional non-spatial clustering approaches, such as the Louvain algorithm, utilize only gene expression data, neglecting spatial context [11]. This limitation often results in incoherent clustering outcomes within tissue sections. In contrast, spatial clustering methods like BayesSpace employ a fully Bayesian statistical framework to infer the spatial distribution of spots, thereby identifying distinct spatial regions and their associated expression patterns [12]. Despite the potential of spatial positional information to enhance clustering accuracy, these methods have yet to achieve optimal performance.

Recent studies have highlighted the effectiveness of graph neural networks (GNNs) in integrating transcript expression with spatial coordinates [6,9,13,14]. GNNs effectively model the local environments surrounding cells, allowing the expression patterns of neighboring cells to influence one another, thus capturing spatial heterogeneity. Through graph structures, information can propagate between nodes, enhancing the model’s understanding of complex relationships between spatial positions and transcript expression [15]. For instance, SpaGCN is an unsupervised clustering algorithm based on graph convolutional networks (GCNs) [16] that integrates gene expression, histological images, and spatial positional information to identify spatial domains. However, SpaGCN is predominantly driven by gene expression, leading to discrepancies between detected regions and actual tissue structures [9].

Furthermore, advanced methods for analyzing SRT data employ graph autoencoders (GAE) or graph contrastive learning (GCL) to learn latent representations of gene expression for spots [15,17–19]. For example, SEDR constructs low-dimensional latent representations of gene expression using deep autoencoders, simultaneously incorporating the corresponding spatial information through variational graph autoencoders [20]. The adaptive graph attention autoencoder method STAGATE learns latent representations by integrating spatial information and gene expression, adaptively capturing similarities between adjacent spots [6]. CCST, a GCL method built on the Deep Graph Infomax (DGI) framework [21,22], utilizes the principle of maximizing mutual information to bring similar views closer while distancing unrelated views. GraphST employs a dual-channel approach using shared-weight methodologies, where one channel reconstructs gene expression based on GAE, and the other is based on the DGI framework for GCL [23]. This dual modeling approach aims to learn latent embeddings of spatial transcriptomics data. Despite their success in single-slice analysis, challenges arise when scaling up to multi-slice data, especially when slices do not perfectly overlap or contain distinct specialized clusters, leading to inaccurate data integration. Additionally, these methods face performance limitations when analyzing multiple SRT datasets comprehensively, as batch effects can obscure the underlying biological signals [7,10].

Several methods and tools have been developed to address the challenges of integrating multiple SRT datasets. These methods are typically designed to mitigate batch effects, align spatial domains, and improve clustering performance across different slices. One such method, STitch3D [24] aligns 2D slices using the Iterative Closest Point (ICP) or PASTE algorithm and constructs a global 3D spatial adjacency graph structure. This model integrates slice- and point-specific effects as well as gene-specific effects into a graph attention autoencoder to mitigate batch effects between slices. Splane [25] uses the Spoint model for deconvolution analysis of SRT data, replacing the original transcript expression with the obtained cell-type composition for downstream analysis. By integrating adversarial learning strategies, Splane effectively eliminates batch effects between slices. STAligner [26] combines STAGATE with a method based on mutual nearest neighbors (MNN) to achieve spatial awareness across multiple SRT datasets. This unified model attempts to construct MNN pairs between slices, but its reliance on MNNs may present issues. In real-world scenarios, slices may not contain the same specialized clusters, such as those collected at different time points. This can lead to incorrect MNN pairings across spatial domains. Furthermore, the computational intensity of searching for MNN pairs may result in missed connections between non-MNN pairs within the same functional cluster. Each of these methods has contributed valuable insights to the field, but they also present challenges related to assumptions of dependence on specific preprocessing steps.

To address these challenges, we propose SpaMask, a dual-masking graph autoencoder with contrastive learning for SRT analysis. Unlike previous approaches such as GAE and GCL, SpaMask employs Masked Graph Autoencoders (MGAE) and Masked Graph Contrastive Learning (MGCL) modules, utilizing a shared graph encoder to integrate MGAE and MGCL for deriving latent representations of gene expression. In the MGAE channel, we implement a node masking mechanism that randomly masks the gene expression of selected spots [27,28]. Under the influence of reconstruction loss, the information propagation mechanism of GNNs compels this channel to leverage the spatial neighbor information of the masked spots to infer the features of the target spot, thereby enhancing the suitability of these features for spatial domain clustering. In the MGCL channel, we utilize an edge masking mechanism to randomly remove certain neighboring edges in the constructed spatial neighbor graph. Driven by contrastive loss, this channel infers potential missing edges from the remaining edges based on spatial proximity and feature similarity [29,30], facilitating tighter embeddings of adjacent nodes in the latent space. This approach ensures that the learned features reflect both spatial proximity and feature similarity. Finally, collective optimization through shared weights enables mutual enhancement of the dual masking.

To evaluate the effectiveness of SpaMask, we conducted comparisons with various existing methods across eight datasets from five different platforms. The results demonstrate that SpaMask exhibits strong competitiveness in terms of clustering accuracy, dispersion, and batch correction capabilities (See Sect A in S1 Text).

## 2. Materials and methods

### 2.1. Dataset description

We used eight datasets from five different platforms, including the 10x Visium platform (human dorsolateral prefrontal cortex [31], human breast cancer [20], and the Sects 1 and 2 of the mouse brain [32]), the ST platform (human melanoma [33]), the Stereo-seq platform (9.5E mouse embryo and mouse olfactory bulb [5]), the osmFISH platform (mouse somatosensory cortex [34,35]), and the MERFISH platform (mouse hypothalamic preoptic regions at Bregma -0.04 mm, -0.09 mm, -0.14 mm, -0.19 mm and Bregma -0.24 mm [36]). A detailed description and data sources are provided in Sect B in S1 Text and S1 Table.

### 2.2. Overview of SpaMask

**Fig 1:**
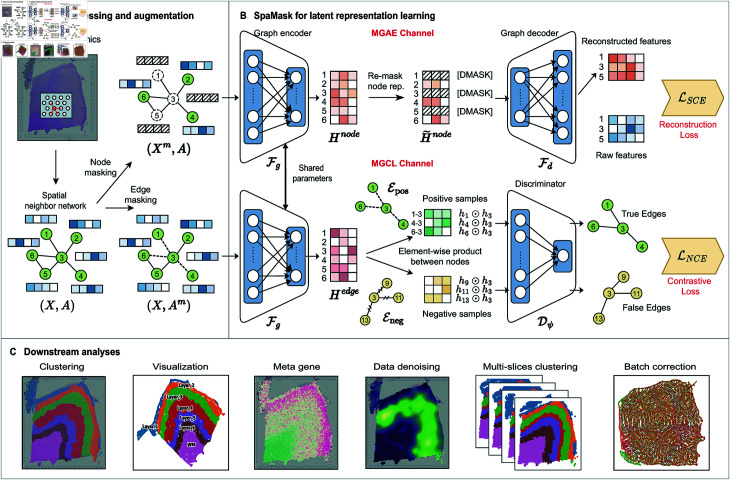
Overview of SpaMask. (A) SpaMask employs two distinct masking techniques to handle the gene expression matrix and spatial topology structure separately. (B) SpaMask integrates Masked Graph Autoencoders (MGAE) and Masked Graph Contrastive Learning (MGCL) modules. MGAE employs node masking to infer missing features based on spatial neighbor information. MGCL applies edge masking to create a contrastive loss framework that tightens embeddings of adjacent nodes based on spatial proximity and feature similarity. (C) The learned latent representations are applied to spatial clustering, trajectory inference, gene expression imputation, and other downstream analytical tasks.

SpaMask begins with preprocessing and augmenting SRT data (Fig 1A). First, the data undergoes filtering and normalization, followed by the selection of highly variable genes to generate the input for the raw transcriptomic dataset. A spatial adjacency graph is then constructed using the spatial coordinates of the spots to capture the relationships among them. Next, two masking strategies are applied: random node masking and random edge masking. The augmented data is processed through two channels MGAE and MGCL (Fig 1B). Finally, the learned latent representations are used for spatial clustering, trajectory inference, gene expression imputation, and other downstream analysis tasks (Fig 1C).

### 2.3. Data preprocessing and spatial graph construction

**Data preprocessing.** The preprocessing steps involve filtering the SRT data to retain only genes expressed in at least 50 spots, with a minimum count of 10 for per spot (Ref. [20]). This ensures that the analysis focuses on genes with sufficient expression levels across multiple spatial regions. The expression matrix is then normalized to account for differences in sequencing depth between spots. Let $X^{\text{raw}}$ represent the raw count matrix. The normalization process adjusts the total counts per spot to a fixed value of $10^{6}$, ensuring comparability across spots. The normalized expression value for each gene *j* in spot *i* is computed as:


$$\begin{array}{ccc} X_{ij}^{\text{normalized}}=\frac{X_{ij}^{\text{raw}}}{\underset{j}{\sum }X_{ij}^{\text{raw}}}\times 10^{6} & & \end{array}$$ (1)

After normalization, the top $N_{hvg}$ highly variable genes are selected based on their variability across samples. Subsequently, the data is scaled to have a mean of 0 and a variance of 1. For denoising tasks, the scaled data is directly used as input. For clustering tasks, dimensionality reduction is further performed using principal component analysis (PCA) [37], where the top $N_{pca}$ principal components are selected as feature inputs. This step reduces data complexity while preserving the most informative features. The final count matrix input is represented as $X\in {\mathbb{R}}^{N_{spot}\times N_{feat}}$, where $N_{feat}$ varies based on the task.

**Spatial Graph Construction.** The key advantage of SRT data lies in its ability to simultaneously capture the spatial distribution of gene expression and the structural information of tissues. According to the “spatial proximity" hypothesis [27], spots that are close in spatial position tend to exhibit some degree of similarity; thus, spatially adjacent spots in tissue sections may have similar gene expression patterns. To effectively capture this similarity, we construct a spatial adjacency graph using spatial coordinates. In this graph, each node represents a spot, and the edges indicate the connections between spatially neighboring spots.

Using the K-Nearest Neighbors (KNN) algorithm, we calculate the Euclidean distances between spots based on their coordinates. For each spot, we select the K nearest neighbors and construct an initial adjacency matrix $A\in {\mathbb{R}}^{N_{spot}\times N_{spot}}$. The matrix is defined as follows: if spot *j* is a neighboring node of spot *i*, then $A_{ij}=1$; otherwise, $A_{ij}=0$.

### 2.4. Data augmentation under dual masking mechanism

In GNNs, node masking and edge masking are crucial mechanisms for enhancing feature learning and clustering performance. Node masking obscures feature nodes, compelling the model to rely on information from neighboring nodes to infer the features of the masked nodes [27,38]. Edge masking randomly obscures certain edges, encouraging the model to learn more robust and meaningful representations from the remaining graph structure, thereby capturing the most expressive relationships within the data [29]. Subsequently, relevant symbols will be defined, followed by a detailed explanation of the masking operations.

The set of nodes representing all spots is denoted as *V*, with the corresponding gene expression represented by *X*. The adjacency relationships between spots are represented by the edge set *E*, based on the constructed adjacency matrix *A*. For the node masking operation, a feature masking rate $\rho_{m}$ is predefined, and nodes are randomly selected from *V* using a random permutation to form the node mask set $V_{m}$, ensuring that the number of masked nodes $\left|V_{m}|=\rho_{m}\times |V\right|$. Consequently, the masked transcript expression matrix $X^{m}$ is defined as follows: if the *i*-th spot satisfies $v_{i}\in V_{m}$, then $X_{i}^{m}$ is replaced with the mask token [MASK], i.e., $X_{i}^{m}=X_{\left[M\right]}$; otherwise, it remains unchanged. Formally, this can be expressed as:


$$\begin{array}{ccc} X_{i}^{m}=\{\begin{array}{ccc} X_{\left[M\right]} & & v_{i}\in V_{m}\\ X_{i} & & v_{i}\notin V_{m} \end{array} & & \end{array}$$ (2)

Similarly, for the edge masking operation, an edge dropout rate $\rho_{d}$ is predefined, and edges are randomly sampled from the edge set *E* using a Bernoulli distribution, resulting in the masked edge set $E_{d}=\{e\in E|\text{Bernoulli}(\rho_{d})=1\}$. Consequently, the masked spatial adjacency matrix $A^{m}$ is defined as follows: for any two spots *i* and *j*, if the edge between them satisfies $e_{ij}\in E_{d}$, then $A_{ij}^{m}=0$; otherwise, it remains unchanged. Formally, this can be expressed as:


$$\begin{array}{ccc} A_{ij}^{m}=\{\begin{array}{ccc} 0 & & e_{ij}\in E_{d}\\ A_{ij} & & e_{ij}\notin E_{d} \end{array} & & \end{array}$$ (3)

### 2.5. Masked Graph Autoencoder (MGAE)

MGAE adopts node masking, allowing the masked spots to rely more on the information provided by adjacent nodes to learn their latent representations. MGAE consists of two components: a graph encoder $F_{g}$ and a graph decoder $F_{d}$.

#### 2.5.1. Graph encoder for latent representation learning under node masking.

Graph Convolutional Networks (GCNs) excel at aggregating and processing information from neighboring nodes. We leverage GCN as the backbone model to construct the graph encoder for learning latent representations. The input to the encoder includes the masked gene expression matrix $X^{m}$ and the unmasked spatial adjacency matrix *A*. The latent representations of masked nodes are inferred through the multi-layer GCN mapping function $F_{g}$, parameterized by *A*. Consequently, the latent representations of all nodes are denoted as $H^{node}\in {\mathbb{R}}^{N_{spot}\times N_{latent}}$, where $N_{latent}$ represents the dimensionality of the latent space. By utilizing information from unmasked neighboring nodes, the GCN learns expressive node representations. The process is formally expressed as:


$$\begin{array}{ccc} H^{node}=F_{g}(X^{m},A;\theta )=Â\text{PReLU}(\text{BN}(ÂX^{m}W_{g}^{0}))W_{g}^{1} & & \end{array}$$ (4)

where $W_{g}^{\left(l\right)}$ represents the weights of the *l*-th layer, and $Â=D^{-1∕2}AD^{-1∕2}$ is the symmetrically normalized adjacency matrix applied to enhance the effectiveness of information propagation. PReLU (Parametric ReLU) is an activation function that introduces a learnable parameter to adjust the slope of the negative half-axis, helping to improve the network’s expressiveness. BN (Batch Normalization) is used to standardize the input, reducing internal covariate shift and improving training stability.

#### 2.5.2. Graph decoder for node features reconstruction.

To enhance the robustness and clustering performance of the autoencoder, we introduce a remasking technique that forces the decoder to extract critical information from unmasked neighboring nodes to reconstruct the raw data. Specifically, for the *i*-th spot $v_{i}\in V_{m}$, its latent representation $H_{i}^{node}$ is replaced by a remask token [DMASK], i.e., ${\tilde{H}}_{i}^{node}=H_{\left[M\right]}$. This design ensures that the decoder can effectively utilize information from neighboring nodes, enabling it to reconstruct the masked nodes more accurately with the assistance of the unmasked neighboring latent representations. Formally, this can be expressed as:


$$\begin{array}{ccc} {\tilde{H}}_{i}^{node}=\{\begin{array}{ccc} H_{\left[M\right]} & & v_{i}\in V_{m}\\ H_{i}^{node} & & v_{i}\notin V_{m} \end{array} & & \end{array}$$ (5)

The decoder takes the remasked latent representation ${\tilde{H}}^{node}$ and the adjacency matrix *A* as inputs. Parameterized by *ϕ*, the decoder serves as the mapping function $F_{d}$ and consists of a linear layer followed by a GCN layer. The computation process is as follows:


$$\begin{array}{ccc} Z=F_{d}({\tilde{H}}^{node},A;\phi )=Â\text{PReLU}(\text{BN}({\tilde{H}}^{node}W_{d}^{0}))W_{d}^{1} & & \end{array}$$ (6)

where PReLU is an activation function, BN is batch normalization and $Z\in {\mathbb{R}}^{N_{spot}\times N_{feat}}$ represents the reconstructed expression data, while $W_{d}^{\left(0\right)}$ and $W_{d}^{\left(1\right)}$ denote the weights of the linear layer and the GCN layer, respectively.

#### 2.5.3. Reconstruction loss function.

To reconstruct the masked features from the given partially observed input features, we utilize the Scaled Cosine Error (SCE) as the reconstruction objective function. The $l_{2}$-normalized cosine error enhances the stability of the embedding representation learning. With a predefined scaling factor *γ*, the similarity between the reconstructed expression data *Z* and the raw input data *X* is calculated exclusively for the set of masked nodes. The mathematical formulation is as follows:


$$\begin{array}{ccc} L_{SCE}=\frac{1}{\left|V_{m}\right|}\underset{v_{i}\in V_{m}}{\sum }{\left(1-\frac{X_{i}Z_{i}^{⊤}}{∥X_{i}∥\cdot ∥Z_{i}∥}\right)}^{\gamma },\;\gamma \geq 1 & & \end{array}$$ (7)

where *γ* is fixed at 2 throughout the experiment to diminish the impact of contributions from simpler samples during training, and $\left|V_{m}\right|$ represents the number of nodes in the masked set.

### 2.6. Masked Graph Contrastive Learning (MGCL)

Edge masking of MGCL is employed to randomly hide certain edges, encouraging the model to capture more robust and expressive relationships within the remaining graph structure. This allows the model to infer the masked edge set from the remaining edges. MGCL consists of two components: a graph encoder $F_{g}$ and a positive-negative edge discriminator *D*.

#### 2.6.1. Graph encoder for latent representation learning under edge masking.

To effectively leverage the transmission of information from neighboring nodes and the complementary nature of local context, we employ a graph encoder with the same structure in both the edge masking and node masking scenarios, sharing weights and optimizing collectively (i.e., $F_{g}(\theta )$). This mutual enhancement through dual masking is significant because, when node masking occurs, the model emphasizes relying on the retained edge structure to infer node features. Conversely, in the case of edge masking, it relies on the retained node information to infer the graph structure.

In this process, the graph encoder $F_{g}$ takes the raw expression matrix *X* and the masked spatial adjacency matrix $A^{m}$ as inputs. According to the graph encoder computation formula (4), we learn the latent representation defined as $H^{edge}=F_{g}(X,A^{m};\theta )$, where $H^{edge}\in {\mathbb{R}}^{N_{spot}\times N_{latent}}$.

#### 2.6.2. Positive and negative edge set construction.

As previously mentioned, contrastive learning typically relies on constructing positive and negative sample pairs. In our work, the set of masked edges $E_{d}$ is explicitly defined as the positive edge set. This choice is motivated by two reasons: first, $E_{d}$ is constructed based on the distances of spatial coordinates, which preserves the spatial proximity among the spots; second, reconstructing the masked edges in the latent space enhances the similarity of representations among adjacent nodes. The negative edge set can be constructed by randomly selecting non-adjacent spots. Given that the total number of spots within a slice far exceeds the number of spatial neighbors, this approach not only ensures randomness but also accelerates computation.

For the construction of the negative edge set based on the positive edge set $E_{\text{pos}}$, we define it as follows. The positive edge set $E_{\text{pos}}$ is equivalent to the masked edge set $E_{d}$, consisting of edges $e_{ij}=(v_{i},v_{j})$ between node $v_{i}$ and its neighbor node $v_{j}$, expressed as:


$$\begin{array}{ccc} E_{\text{pos}}=\{e_{ij}|e_{ij}\in E_{d}\} & & \end{array}$$ (8)

To construct the negative edge set $E_{\text{neg}}$, we ensure that the number of negative edges $n_{i}$ for each node $v_{i}$ equals the number of positive edges $\left|N(v_{i})\right|$ in the positive edge set $E_{\text{pos}}$. Specifically, we randomly select $n_{i}$ nodes $v_{k}$ from the node set *V*, ensuring that these nodes do not belong to $N(v_{i})$, meaning that the negative edges do not overlap with the positive edges. The negative edge set $E_{\text{neg}}(v_{i})$ for node $v_{i}$ can be expressed as:


$$\begin{array}{ccc} E_{\text{neg}}(v_{i})=\{e_{ik}=(v_{i},v_{k})|v_{k}\in V\setminus N(v_{i}),|E_{\text{neg}}(v_{i})|=n_{i}\} & & \end{array}$$ (9)

The final negative edge set is the union of the negative edge sets for all nodes $v_{i}$, expressed as:


$$\begin{array}{ccc} E_{\text{neg}}=\underset{v_{i}\in V}{⋃}E_{\text{neg}}(v_{i}) & & \end{array}$$ (10)

Through these steps, we effectively construct the positive and negative edge sets, ensuring the mutual exclusivity of negative edges with respect to positive edges in the node neighborhoods, while providing reasonable positive and negative sample pairs for subsequent contrastive learning tasks.

#### 2.6.3. Discriminator of MGCL.

The discriminator aims to differentiate between positive edges (i.e., edges that genuinely exist but are masked) and negative edges (i.e., nonexistent edges) within a graph structure. Given a node pair $\left(v_{i},v_{j}\right)$, the discriminator takes their latent feature representations as input and predicts the likelihood of a connection between $v_{i}$ and $v_{j}$ existing in the original graph structure using a feedforward neural network.

The discriminator function $D_{\psi }(h_{i},h_{j})$ processes $h_{i}$ and $h_{j}$ through concatenation, Hadamard product, or other composite representations (e.g., $h_{i}⊙h_{j}$). Subsequently, it applies a series of transformations, such as linear layers and activation functions, resulting in a scalar output representing the probability of the edge being real. This discriminator model is capable of learning complex edge structures, making it a valuable tool for validating learned graph embeddings or serving as an adversary to enhance the quality of generated edges in generative graph models. The inference process involves computing:


$$\begin{array}{ccc} D_{\psi }(h_{i},h_{j})=\text{Sigmoid}(W_{2}\cdot \text{ReLU}(W_{1}\cdot (h_{i}⊙h_{j}))+b_{1})+b_{2}) & & \end{array}$$ (11)

where $W^{0}$ and $W^{1}$ are the weight matrices of the first and second layers, respectively, and $b_{0}$ and $b_{1}$ are the corresponding bias terms. During inference, higher values of $D_{\psi }(h_{i},h_{j})$ indicate that the edge relationship between $v_{i}$ and $v_{j}$ is more likely to be authentic, while lower values suggest otherwise. This mechanism enables nuanced understanding and modeling of graph edge relationships, capturing both linear and nonlinear dependencies among nodes.

#### 2.6.4. Contrastive loss function.

Edge Noise Contrastive Estimation (NCE) [39] implicitly enhances mutual information between positive samples and the target variable by contrasting the probability distributions of positive and negative samples (See Sect C in S1 Text). By maximizing the probability of positive samples, the model effectively captures the dependencies among them, while minimizing the probability of negative samples reduces the influence of noise or irrelevant data. NCE loss reframes the complex problem of mutual information estimation into a binary classification task, where contrastive learning is used to distinguish positive samples from negative ones. In a self-supervised learning setup, this method drives the model to approximate the true distribution of positive samples and maximize the contrast between positive and negative examples, thereby enhancing mutual information. The NCE loss is calculated as follows:


LNCE=− (1|Epos|∑(u,v)∈Epos log ⁡ Dψ(Huedge,Hvedge)+1|Eneg|∑(u′,v′)∈Eneg log ⁡ (1−Dψ(Hu′edge,Hv′edge))) (12)

### 2.7. Overall loss function of SpaMask

The final learning objective is a weighted sum of the reconstruction loss $L_{SCE}$ and the contrastive loss $L_{NCE}$. The parameter *λ* controls the trade-off between these two losses, balancing the reconstruction of masked features and the discrimination of positive and negative samples. The overall objective function is expressed as:


$$\begin{array}{ccc} L=(1-\lambda )L_{SCE}+\lambda L_{NCE} & & \end{array}$$ (13)

where *λ* ∈ [ 0 , 1 ]  is a hyperparameter that adjusts the contribution of each loss term.

### 2.8. Extending SpaMask to integrate multiple slices

Unlike traditional single-slice clustering algorithms, SpaMask integrates multiple slices by focusing on both vertical and horizontal slice integration (S1 Fig).

In vertical continuous slice integration, SpaMask utilizes an iterative closest point (ICP) algorithm for multi-slice alignment [40], registering spatial points to accurately align multiple slices. The alignment details are provided in Sect D in S1 Text. After aligning the slices, we introduced a z-axis to represent inter-slice distances, using the default distance between two spots, and constructed a 3D neighborhood graph connecting all spots across slices based on these coordinates [24].

For horizontal slice integration, we referred to the horizontal alignment method of stGCL [41] and adapted it to suit SpaMask. Specifically, adjacent tissue slices are translated and aligned along the *x* and *y* axes. Given two slices, the rightmost point in the left slice (Batch1) and the leftmost point in the right slice (Batch2) are considered as points on the cutting surface edge. The average *y* coordinates of the two slice edge points, $\left\{{ȳ}_{1},{ȳ}_{2}\right\}$, are computed. Meanwhile, the maximum *x* coordinate of all points in Batch1, $\max(x_{1})$, and the minimum *x* coordinate of all points in Batch2, $\min(x_{2})$, are determined. Finally, Batch1 is fixed in position, and Batch2’s coordinates are adjusted according to the calculated offsets, $\Delta_{x}=\max(x_{1})-\min(x_{2}),\Delta_{y}={ȳ}_{1}-{ȳ}_{2}$, to achieve horizontal integration.

### 2.9. Baseline methods

**SpaMask and SpaMask_D:** By default, **SpaMask** applies PCA on the obtained highly variable genes, selecting the top 200 principal components as input features. To demonstrate the capability of our method in gene denoising, we define **SpaMask_D**, which directly uses highly variable genes as input and has the same model architecture as SpaMask. Finally, SpaMask_D uses the reconstructed data as the denoised gene expression for downstream denoising analysis.

To evaluate the performance of our proposed method, we selected a range of representative state-of-the-art methods. For spatial domain identification in single slices, we chose two methods that leverage histological image features combined with spatial expression and coordinate information: SpaGCN [9] and DeepST [42]. Additionally, we included two reconstruction-based approaches: SEDR [20] which utilizes a deep autoencoder structure, and STAGATE [6] which employs an adaptive attention mechanism. We also incorporated two contrastive learning-based methods: CCST [22], based on the Deep Graph Infomax (DGI) approach, and GraphST [23] which uses DGI while also reconstructing the original features. Finally, we included DiffusionST [43], a method based on a diffusion model.

For spatial domain identification across multiple slices, we adopted STAligner [26], Splane [25], STitch3D [24], SPIRAL [44] and stGCL [41] as baseline methods. The configurations of each method are detailed in Sect E in S1 Text and S2 Table.

### 2.10. Evaluation criteria

We evaluated the accuracy of spatial domain identification using the Adjusted Rand Index (ARI) [45] and Accuracy (ACC)[7], where higher values, approaching 1, indicate better performance. The Discreteness Index (DIS) [7] was used to assess the degree of discreteness within the identified spatial domains; smaller DIS values, approaching 0, signify fewer scattered spots and clearer domain boundaries. We used Moran’s *I* and Geary’s *C* to assess the spatial autocorrelation of specific genes, revealing their spatial expression patterns. For multi-slice batch correction, we employed the F1LISI metric [46] to describe the batch correction ability, where a value close to 1 indicates strong correction. Detailed definitions of these metrics are provided in Sect F in S1 Text.

## 3. Results

### 3.1. Experiment settings

In data preprocessing, the default selection is 2000 highly variable genes, and for PCA feature extraction, the first 200 PCs are chosen as input features. If the number of genes is fewer than the number of PCs, the original number of genes is preserved (S2 Fig). For the SpaMask model, a learning rate of 0.001 and a weight decay rate of 2e-4 were used on all datasets. The Adam optimizer was employed for optimization. The shared encoder consisted of two GCN layers, with output dimensions of 512 and 256, respectively. The reconstruction comprised of one linear layer and one GCN layer, while the discriminator consisted of two linear layers. During the clustering evaluation, the obtained encodings were subjected to PCA to extract the top 30 principal components (S3 Fig), followed by k-means clustering to obtain the final clustering results. For all baselines, default parameters from the original papers were used, and all experiments were conducted on an NVIDIA GeForce RTX 3090.

**Selection of parameters *λ*, $\rho_{m}$ and $\rho_{d}$.** In SpaMask, we conduct experiments on multiple datasets, determining the default node mask rate $\rho_{m}=0.3$ and the default edge mask rate $\rho_{d}=0.4$. For high-resolution datasets (e.g., FISH), we recommend increasing the edge mask rate to 0.5. The default weight factor for controlling the reconstruction loss and contrastive loss is set to *λ* = 0 . 7. The detailed experiments and results for parameter selection can be found in Sect G in S1 Text and S4 Fig.

### 3.2. Leveraging SpaMask for improved clustering and structural insights in DLPFC

**Fig 2:**
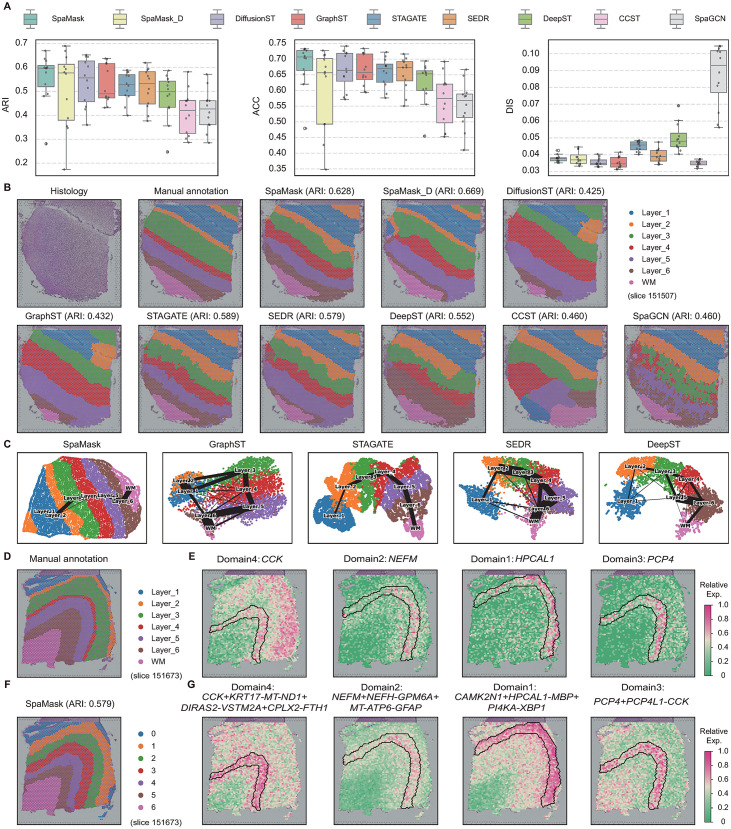
SpaMask enhances tissue structure identification in human DLPFC tissue. (A) Boxplots of ARI (left), ACC (middle) and DIS (right) scores to all DLPFC slices. (B) Tissue image, manually annotated layer structures, and spatial domains detected by nine methods on slice 151507. (C) UMAP visualization and PAGA graph generated by SpaMask, GraphST, STAGATE, SEDR and DeepST embeddings respectively on slice 151507. (D) Manually annotated layer structures of slice 151673. (E) Spatial expression patterns of SVGs detected by SpaMask on slice 151673. (F) Spatial domains detected by SpaMask. (G) Spatial expression patterns of meta-genes detected by SpaMask.

To comprehensively and quantitatively assess SpaMask’s performance in spatial domain identification, we applied the method to a human dorsolateral prefrontal cortex (DLPFC) dataset generated on the 10x Visium platform. This dataset comprises 12 cortical slices with spatial expression data. Using morphological characteristics and gene markers, Maynard et al [31]. manually annotated the DLPFC layers and the white matter (WM) region. We evaluated SpaMask’s clustering accuracy against seven baseline spatial clustering methods and a denoised variant of SpaMask, using the ARI, ACC, and DIS as evaluation metrics (Fig 2A) [7]. Higher ARI and ACC values indicate better clustering precision, while a lower DIS value reflects fewer dispersed spots, suggesting a more continuous spatial domain. The results demonstrated that SpaMask achieved superior clustering performance across all 12 tissue slices, with a notable median ARI of 0.596, representing a 6.9% and 6.5% improvement over the median ARIs of STAGATE (0.527) and SEDR (0.531), respectively. Additionally, the denoised SpaMask variant attained a median ARI of 0.56.

For instance, in slice 151507 (Fig 2B), SpaMask accurately delineated distinct layer boundaries and exhibited strong concordance with manually annotated spatial domains, achieving optimal clustering accuracy (ARI = 0.628, ACC = 0.730). Furthermore, SpaMask-identified spatial domains displayed lower spot discreteness (DIS = 0.036). In contrast, SpaGCN, which leverages tissue image features, struggled to effectively capture meaningful image characteristics, failing to distinguish layers 4, 5, and 6 and exhibiting high spot mixing and the highest observed discreteness (DIS = 0.101). Although CCST, which maximizes mutual information between graph nodes, achieved the lowest discreteness (DIS = 0.034), it was unable to differentiate accurately between cortical and WM layers. Other methods, such as STAGATE (ARI = 0.589, ACC = 0.709) and SEDR (ARI = 0.579, ACC = 0.702), showed partial success in identifying certain layers but struggled with accuracy and layer thickness representation. In comparison, SpaMask effectively identified the anticipated cortical layer structure.

In further analyses, we employed UMAP visualization [47] to examine the role of expressive embeddings in domain identification (Fig 2C and Sect H in S1 Text). The UMAP plot generated from SpaMask embeddings revealed spatially continuous trajectories across layers (from layer 1 to layer 6 and WM) [48], reflecting functional similarities between adjacent cortical layers and indicating a near-linear developmental trajectory. The clear layer separation observed in the UMAP plot corroborated that SpaMask embeddings effectively capture spatial domain characteristics. In contrast, UMAP plots from baseline methods showed irregular patterns. For example, GraphST visually displayed distinct inter-layer separation with low discreteness, but its embeddings appeared disorganized due to GraphST’s clustering refinement function, which relies on neighboring spot information and limits trajectory analysis of embeddings. DiffusionST presented similar UMAP patterns to GraphST, as detailed in S5 and S6 Figs.

Following a procedure similar to that used in SpaGCN, we identified spatially variable genes (SVGs) enriched in each spatial domain [9]. Differential expression (DE) [49] analysis was conducted on spots within the target domain and adjacent domains [50], selecting genes with FDR-adjusted *P* values  <  0.05 as SVGs. A total of 76 SVGs were identified on slice 151673, with domain 0 containing 68 SVGs, while domains 1, 2, and 6 each contained 1 SVG, and domain 4 contained 5 SVGs. Different colors represent the relative expression levels of these genes. For example (Fig 2E), *NEFM* was enriched in domain 2 (layer 3), *PCP4* in domain 3 (layers 4 and 5), and *HPCAL1* in domain 1 (layer 2), aligning with previous findings [51]. When expression patterns within specific neuronal layers could not be clearly identified using single gene markers, we constructed metagenes composed of multiple genes to characterize domain-specific expression. For instance (Fig 2G), *CCK* exhibited weak enrichment across layers 2, 3, and 6 with limited spot numbers. By combining genes such as *KPT17*, *DIRAS2*, and *CPLX2* into a metagene, we clarified the expression pattern in target domain 4 (layer 5). We further observed that these SVGs were transferable across several other tissue slices (See Sect I in S1 Text and S7 Fig).

### 3.3. SpaMask enhances denoising in layer-specific gene expression visualization

The raw SRT data is susceptible to noise from sequencing techniques, which hampers the accurate representation of spatial expression patterns and affects downstream analyses, such as cell clustering, differential expression analysis, and cell trajectory inference. To address this issue, we applied the denoised variant of SpaMask to the DLPFC dataset. By constructing a denoised gene expression matrix, we effectively reduced noise in the raw data and enhanced the identification rate of spatial gene expression patterns.

**Fig 3:**
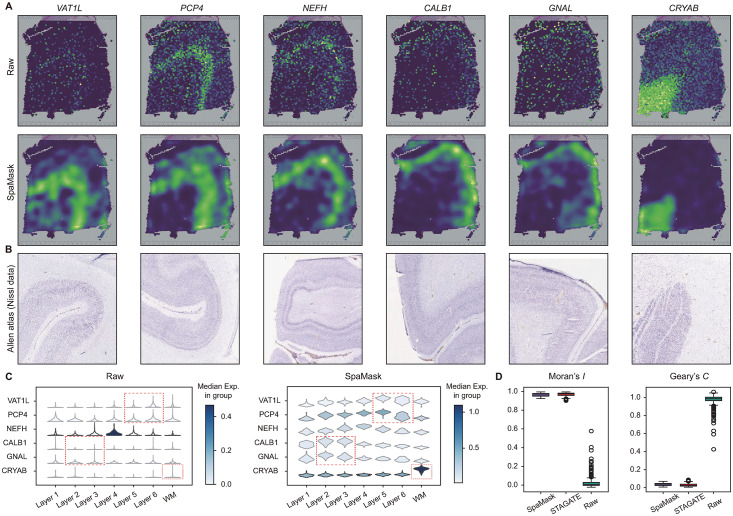
SpaMask enhances spatial patterns of layer-specific marker genes in the DLPFC dataset. (A) Visualization of the raw and SpaMask-denoised spatial expression data for six layer-specific marker genes on slice 151674. (B) Nissl images sourced from the publicly available Allen Human Brain Atlas. (C) Violin plots showing the raw and denoised expression levels of layer-specific marker genes. Red boxes highlight the cortical layers corresponding to each layer-specific marker gene. (D) The Moran’s *I* and Geary’s *C* indices for the top fifty differentially expressed genes from SpaMask, STAGATE, and raw data.

For example, on slice 151674 of the DLPFC, we compared the expression profiles of six layer marker genes (*VAT1L*, *PCP4*, *NEFH*, *CALB1*, *GNAL*, *CRYAB*) between the raw data and SpaMask-denoised data (Fig 3A). In the raw data, the within-layer patterns of these marker genes appeared dispersed, while the denoised data more clearly revealed gene enrichment across layers, consistent with the Nissl data published by the Allen Human Brain Atlas [52] (Fig 3B). Additionally, we observed significant enrichment of *PCP4* in layer 5, aligning with previous research findings [31,51]. Violin plots (Fig 3C) further show that the denoised spatial expression patterns closely match the manually annotated tissue structure (S8 Fig).

We also evaluated the spatial autocorrelation of specific genes using Moran’s *I* and Geary’s *C* indices to reveal their spatial expression patterns (Fig 3D). Specifically, we selected domains with more than three spots and conducted differential expression analysis to rank the top 100 significantly expressed genes. Then, using the constructed spatial adjacency matrix, we defined each spot’s spatial relationships with its neighbors to calculate Moran’s *I* and Geary’s *C* indices (See Sect F in S1 Text). Moran’s *I* values range from –1 to  + 1, with positive values indicating positive spatial autocorrelation and negative values indicating negative correlation. Geary’s *C* values range from 0 to 2, with values below 1 indicating higher similarity within neighborhoods, while values above 1 indicate greater differences. We compared the performance of SpaMask and STAGATE-denoised data against the raw data in terms of Moran’s *I* and Geary’s *C* indices, and the results show that SpaMask significantly reduced noise while preserving spatial expression patterns.

### 3.4. Advancing structural analysis in breast cancer and melanoma with SpaMask

**Fig 4:**
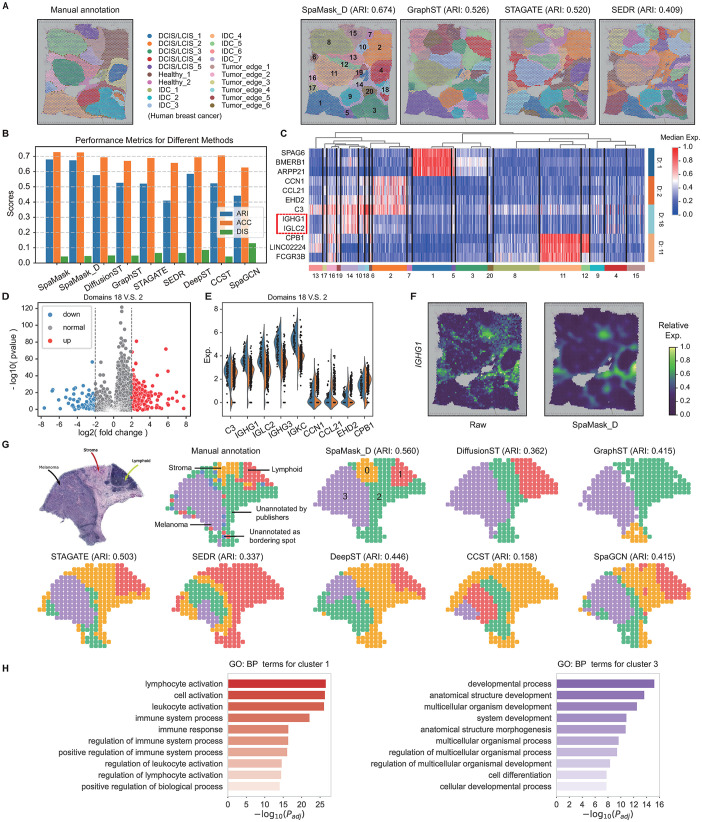
SpaMask improves the identification of known tissue structures in human breast cancer and melanoma tissues. (A) Manually annotated layer structures of human breast cancer tissue (left) alongside spatial domains detected by SpaMask, GraphST, STAGATE, and SEDR (right). (B) A bar chart illustrating the clustering accuracy of various methods on breast cancer, measured using ARI, Accuracy, and Discreteness scores. (C) Heatmap displaying the expression of structural domains for the top three DEGs from domains 1, 2, 11, and 18. (D) and (E) show the volcano plots of DEGs between domain 18 (tumor edge) and domain 2 (healthy), along with the differential expression analysis of specific genes. (F) *IGHG1* serves as a marker gene for displaying the raw and denoised spatial expression. (G) Tissue image, manually annotated layer structures, and spatial domains detected by various methods on human melanoma tissue. (H) Top ten key GO:BP terms for cluster 1 (lymphoid, left) and cluster 3 (melanoma, right).

We conducted a comprehensive evaluation of SpaMask’s performance in the 10x Visium human breast cancer dataset, which was meticulously annotated by Xu et al. [20], includes 20 regions such as DCIS/LCIS, healthy tissue, invasive ductal carcinoma (IDC), and low-grade tumor margins (Fig 4A). While DeepST and SpaGCN exhibited satisfactory performance in identifying major tissue regions through histological information, they struggled with defining spatial domain boundaries, resulting in ambiguities and a substantial number of outliers, with dispersions recorded at DIS = 0.085 and DIS = 0.13, respectively (S9 Fig). Both STAGATE and SEDR faced challenges in accurately identifying IDC regions (IDC 2/4/5), while GraphST misidentified the Healthy1 region, segmenting it into multiple small clusters. In stark contrast, SpaMask achieved the highest clustering performance independent of histological images (ARI = 0.674), showcasing distinct tissue boundaries and fewer outliers (DIS = 0.045), whereas all other methods’ ARI values below 0.6 (Fig 4B).

To delve deeper into the heterogeneity of tumor regions, we analyzed the top three differentially expressed genes within IDC (Cluster 1), healthy tissue (Cluster 2), tumor margins (Cluster 18), and DCIS/LCIS (Cluster 11). A heatmap was generated to illustrate their expression patterns, highlighting significant heterogeneity across the clusters (Fig 4C). For example, the genes *IGHG1* and *IGLC2* were predominantly enriched in Cluster 18, while *CCN1* and *CCL21* showed significant enrichment in Cluster 2. Additionally, a differential expression analysis comparing Clusters 18 and 2 revealed 282 significantly differentially expressed genes ( | *log*2*FoldChange* | ≥ 2 and *P* < 0 . 05) (Fig 4D), with violin plots illustrating the expression distribution of nine high-ranking DEGs (Fig 4E). Related research indicates that the high expression of *IGHG1* is associated with pathological processes such as tumor cell proliferation and migration, facilitating the malignant progression of breast cancer by activating the AKT pathway [53]. We also showcased the denoising capabilities of SpaMask (S9 Fig). The post-denoising gene expression data provided a clearer representation of the enrichment and spatial expression patterns of layer marker genes. For instance, the denoised data indicated substantial enrichment of *IGHG1* in clusters associated with tumor edges (Clusters 10, 14, 16, and 18), which closely aligned with the manually annotated tumor edge regions (Fig 4F).

This expanded assessment of SpaMask on human melanoma dataset from the ST platform offers further insight into its spatial domain identification capabilities. Thrane et al. [33] manually annotated three distinct regions—melanoma, stroma, and lymphatic tissue—alongside an unannotated area (Fig 4G), forming four domains used to evaluate spatial domain identification. SpaMask achieved the highest clustering accuracy, clearly delineating melanoma, stroma, and lymphatic tissue regions, whereas other methods encountered challenges, especially in distinguishing the stroma region. Due to the sparse, low-density characteristics of the human melanoma dataset, CCST, with its four-layer GCN backbone, displayed pronounced over-smoothing, resulting in the lowest performance (S10 Fig).

To perform functional enrichment analysis on Clusters 1 and 3 using the Gene Ontology: Biological Process (GO:BP) database [54], we first conducted differential gene expression analysis for these clusters with thresholds of $|log_{2}(FoldChange)|\geq 2$ and *P* < 0 . 05. Subsequently, under the same conditions, the gene expressions interpolated by SpaMask yielded a greater number of GO terms. Cluster 1, identified as part of the lymphatic family, exhibited 10 GO terms primarily associated with alterations in cellular morphology, behavior, and the modulation of immune responses. In contrast, Cluster 3, corresponding to the melanoma family, was enriched in terms related to biological regulation and metabolic processes (Fig 4H).

### 3.5. Achieving robust spatial domain identification with SpaMask across diverse transcriptomics platforms

**Fig 5:**
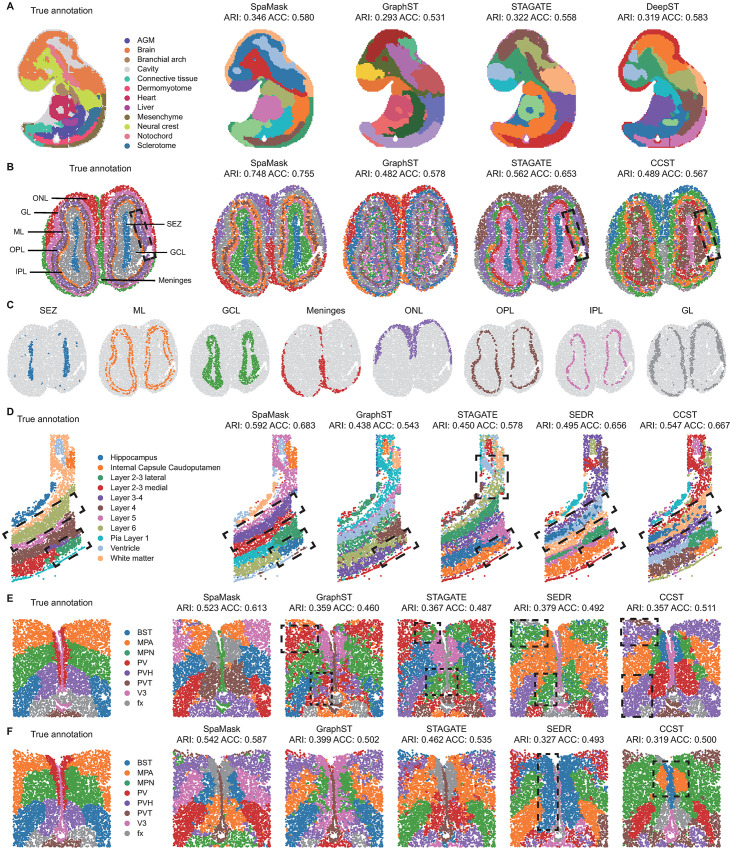
SpaMask performs spatial domain identification across various SRT datasets generated on different platforms. (A) and (B) show the true annotations and clustering results from different methods for mouse embryo and mouse olfactory bulb, respectively, generated using the Stereo-seq platform. (C) Visualization of spatial domains identified by SpaMask in the mouse olfactory bulb data. (D) Visualization of the mouse somatosensory cortex dataset generated by the osmFISH platform, along with the spatial domains identified by various methods. (E) and (F) are visualizations of two specific slices of the mouse hypothalamic preoptic area, located at Bregma –0.04 mm and –0.09 mm, respectively, generated by the MERFISH platform.

With the rapid advancements in SRT technology, various platforms have emerged, making it essential to validate SpaMask’s scalability and robust spatial domain identification across datasets generated from different platforms. A detailed description of all dataset used in this study and their sources can be found in Sect B in S1 Text and S1 Table. We first applied SpaMask to high-resolution spatial transcriptomics datasets from the Stereo-seq platform, including the 9.5E mouse embryo (Fig 5A) and the mouse olfactory bulb (Fig 5B) [5]. Although SpaMask, along with GraphST and STAGATE, struggled to accurately identify the liver region in the mouse embryo [55], it uniquely succeeded in identifying the full forebrain, hindbrain, and dermomyotome regions, achieving higher clustering accuracy (ARI = 0.346).

For the Stereo-seq mouse olfactory bulb dataset, we used the annotations provided in the study by Nie et al. [5,7,17,20], including the subependymal zone (SEZ), mitral layer (ML), granule cell layer (GCL), meninges, olfactory nerve layer (ONL), outer plexiform layer (OPL), inner plexiform layer (IPL), and glomerular layer (GL). SpaMask effectively identified these layered structures in alignment with the annotated regions, producing more refined boundaries compared to other methods. GraphST exhibited spot mixing across domains, while STAGATE and CCST were unable to distinguish the ML and OPL regions. SpaMask demonstrated superior performance in delineating distinct regions within the olfactory bulb (Fig 5C).

We further applied SpaMask to non-gridded datasets from the osmFISH platform, which represent the mouse somatosensory cortex [34,35], with each layer color-coded. This dataset contains only 33 genes, significantly fewer than the number of genes in platforms like 10x Visium and Stereo-seq and below the default PCs setting (200). Therefore, during preprocessing, SpaMask did not select highly variable genes or apply linear dimensionality reduction methods (e.g., PCA or SVD) but directly used the normalized gene expression as input features. The results show that SpaMask outperforms other methods (e.g., GraphST, STAGATE, and SEDR) on this dataset. Other methods exhibited various spatial domain partitioning issues. For example, GraphST, STAGATE, and SEDR failed to distinguish between lateral and medial regions of layers 2-3; STAGATE further segmented white matter into multiple smaller regions, and SEDR and CCST split layer 6 into two subregions. Only SpaMask accurately identified these layers and achieved the highest clustering performance.

Finally, we tested SpaMask’s spatial domain identification on two slices of the mouse hypothalamic preoptic area from the MERFISH platform [36], located at Bregma-0.04 mm and Bregma-0.09 mm. Each slice comprises eight domains with 155 genes, processed similarly to the osmFISH dataset. At Bregma-0.04 mm (Fig 5E), GraphST, STAGATE, and SEDR merged the MPA and PVH regions, failing to distinguish them, while CCST incorrectly combined the MPA and BST regions. Only SpaMask (ARI = 0.523, ACC = 0.613) successfully separated these regions, while all other methods showed ARI scores below 0.5. At Bregma-0.09 mm (Fig 5F), SEDR and CCST produced inaccurate PV region thickness, failing to delineate it properly. Overall, SpaMask demonstrated robust scalability and superior clustering accuracy across various platforms, consistently outperforming existing methods. SpaMask also achieves the best performance on the anterior dataset of the complex mouse brain’s section_1 from the 10x Visium platform, as shown in (S11 Fig).

### 3.6. Integrating multiple DLPFC slices with SpaMask to mitigate inter-slice batch effects

**Fig 6:**
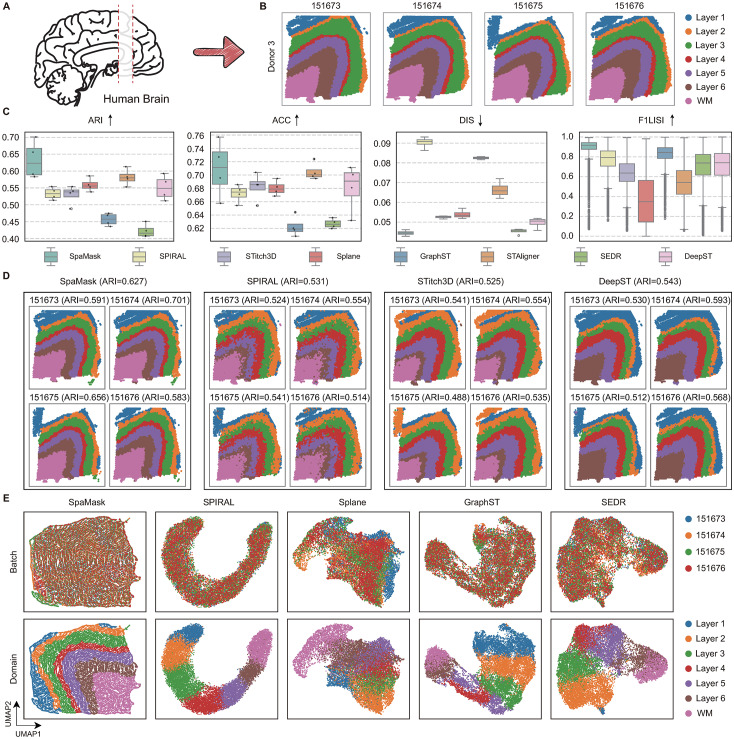
SpaMask effectively alleviates batch effects across continuous slices on the human DLPFC tissue. (A) Adapted from an open-source image available at Openclipart (https://openclipart.org/detail/38533/brain-side-cutaway). (B) Four consecutive slices (151673–151676) from Donor 3 with annotated cortical layers. (C) Box plots comparing SpaMask and various methods (SPIRAL, STitch3D, Splane, GraphST, STAligner, SEDR, and DeepST) across four metrics: ARI, ACC, DIS, and F1LISI. (D) Spatial domains identified by SpaMask, SPIRAL, STitch3D, and DeepST on Donor 3 slices, showing effectiveness in domain detection. (E) UMAP embeddings with color-coded batches (top) and cortical layers (bottom) for SpaMask and comparison methods, highlighting SpaMask’s ability to address batch effects while preserving domain structure across layers.

The human DLPFC tissue dataset includes samples from three independent donors, each represented by four slices. To evaluate SpaMask’s effectiveness in multi-slice dataset integration while maintaining interlayer domain structures and eliminating batch effects, we applied it to a single donor (donor 3, comprising consecutive slices 151673-151676, Fig 6A and 6B) [26,31].

In terms of clustering performance (Fig 6C), STAligner, Splane, and DeepST performed comparably, achieving high ARI and ACC values across consecutive slices, although DeepST missed a layer within the spatial domains. To further assess multi-slice integration correction, we employed the F1LISI metric (See Sect F in S1 Text) [46], where values close to 1 indicate strong correction. Splane yielded the lowest F1LISI score, as shown in its UMAP batch and domain visualizations (Fig 6D and 6E); it failed to eliminate inter-slice differences, resulting in distinct, disorganized domains that highlight its limited batch correction capacity across slices. This constraint likely stems from the inherent limitations of simple adversarial techniques, which improve clustering accuracy across slices but remain fundamentally limited. While STAligner establishes inter-slice MNN pairs that effectively reduce batch effects in single-cell datasets, this method proves less suitable for SRT data, often misaligning MNN pairs across domains and missing intra-domain pairs.

SPIRAL, which incorporates a gradient reversal layer (GRL) to optimize adversarial processes, effectively addresses batch issues by dividing learned latent representations into two parts: one that captures slice-specific differences and another that maximizes inter-slice mixing, resulting in strong batch correction. However, this approach also leads to increased discreteness (DIS mean  >  0.09), with considerable mixing and discontinuities in spatial domains. Although SEDR shows lower clustering accuracy and batch correction, it achieves very low discreteness (mean DIS  <  0.05) due to its use of a deep graph autoencoder that learns from neighboring node embeddings, which supports clustering and aligns closely with SpaMask’s principles.

SpaMask outperformed existing methods in clustering performance; after integrating the slices, it achieved a clustering ARI of 0.627 across all four slices, significantly surpassing other methods. The identified thickness of Layers 2 and 4 closely corresponded to cortical annotations. With its dual-masking complementary enhancement strategy, SpaMask leverages information from both neighbors and masked neighbor relations, resulting in optimal spot continuity and the lowest discreteness level (mean DIS of 0.044). For batch correction, SpaMask achieved a median F1LISI score of 0.918, outperforming all other methods, which scored below 0.9 (Fig 6C). In UMAP visualizations of batch and identified domains, SpaMask successfully integrated slices while preserving interlayer domain structures, significantly mitigating inter-slice batch effects (Fig 6D and 6E). SpaMask is also capable of integrating multiple vertical slices, such as the five slices from different positions of the mouse hypothalamic preoptic region (S12 Fig); it can also integrate horizontal slices, such as the anterior and posterior slices of the mouse brain (S13 Fig).

### 3.7. Ablation studies

We plan to conduct ablation experiments from the following three perspectives.

(1) To assess the effectiveness of applying two masking methods to the two channels of a shared-weight encoder in SpaMask, we designed the first variant, termed One Channel Combined (One Ch, Combine). This variant inputs the masked gene expression matrix $X^{m}$ and the masked spatial adjacency matrix $A^{m}$ into the graph encoder $F_{g}$ to reconstruct the features of the masked spots and infer the deleted edges.
(2) To explore the effectiveness of the dual masking mutual enhancement, we designed seven variants based on the presence or absence of masking and the masking strategies employed. The first variant is a dual-channel without masking (Dual Ch, W/O M), similar to a single-channel without masking. The second and third variants are under the dual-channel setup, where we study the impact of each masking strategy by removing the node or edge masking strategies: dual-channel without node masking (Dual Ch, W/O Node M) and dual-channel without edge masking (Dual Ch, W/O Edge M). The fourth and fifth variants investigate the performance with and without masking under the MGAE setup: masked MGAE node channel (MGAE Ch, W/ M) and unmasked MGAE node channel (MGAE Ch, W/O M). The remaining two variants evaluate the effectiveness of deleting edge relationships in the MGCL edge channel: masked MGCL edge channel (MGCL Ch, W/ M) and unmasked MGCL edge channel (MGCL Ch, W/O M).
(3) We developed two variants focusing on the selection of positive and negative edge sets constructed within the edge pool. For the positive edge set, we implemented Remaining Positive Edge Selection (Pos Sel, Remain), wherein the positive edge set $E_{\text{pos}}$ in SpaMask corresponds to the masked edge set $E_{d}$; this variant utilizes the remaining edge set $E\setminus E_{d}$ as the positive edge set. For the negative edge set, we employed a random sampling method termed Random Negative Edge Selection (Neg Sel, Random).

**Fig 7:**
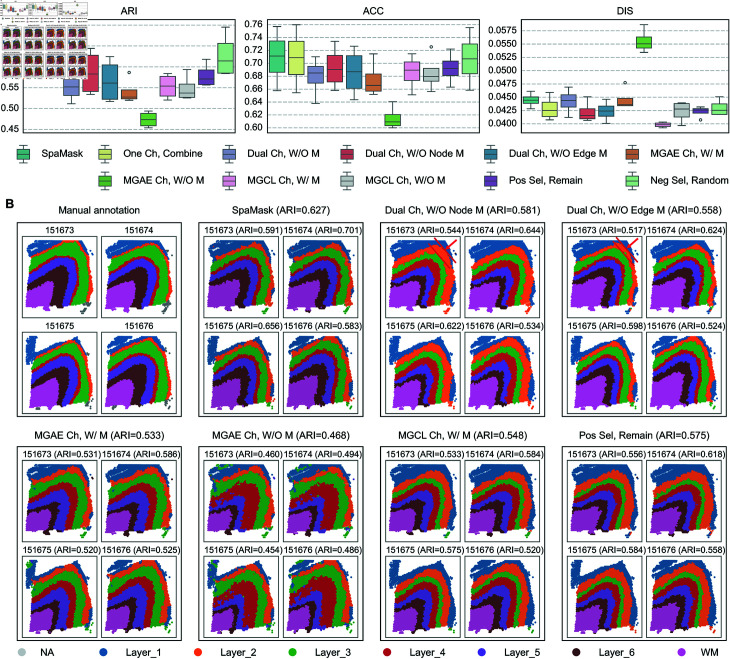
Ablation studies of SpaMask components on human DLPFC Donor 3 data from 10x Visium. (A) The box plot shows ARI, ACC, and DIS metrics, comparing SpaMask configurations to assess component contributions. (B) The spatial domain identification results for Donor 3 emphasize the cortical layer structures identified by SpaMask under different configurations, including dual-channel without node masking or without edge masking, MGAE Channel with/without Mask, MGCL Channel with Mask and Positive Selection with Remain.

**Experimental Results.** We evaluated SpaMask and its variants on the Donor3 data from the DLPFC dataset, the mouse olfactory bulb data from the Stereo-seq platform, the mouse somatosensory cortex data from the osmFISH platform, the mouse hypothalamic preoptic area data from the MERFISH platform at Bregma -0.04 mm, and the human melanoma data from the ST platform. The boxplot (Fig 7A) and the Table 1 comprehensively assess the performance of SpaMask and its variants across multiple datasets. The dual masking mechanism in SpaMask demonstrated superior ARI and ACC performance across various datasets compared to the single-channel combination method (One Ch, Combine), which, in turn, generally outperformed variants lacking one or both masking mechanisms. For example, the Dual Ch, W/O M variant, which lacks both node and edge masking in the dual-channel setting, exhibited significantly lower ARI and ACC metrics across all datasets. These findings highlight the effectiveness of the mutual enhancement achieved by the dual masking mechanism in improving clustering performance.

The random selection variant for negative edge sets (Neg Sel, Random) ranks second in terms of accuracy while exhibiting lower dispersion than SpaMask. This reduced dispersion likely arises from the low probability of randomly sampling negative nodes, which significantly outnumber their neighboring nodes, thereby exerting minimal impact on clustering performance. As shown in Table 1, although Neg Sel, Random performed comparably to SpaMask across other platforms, its performance on the ST platform using human melanoma data (with 293 spots) was significantly inferior to that of SpaMask. The Pos Sel, Remain variant utilized the retained edge set as the positive edge set, effectively reducing to a method that masks only the nodes without applying any masking to the edge set, thus reflecting a difference in the size of the spatial nearest neighborhoods.

Next, we investigated the impact of individual masking mechanisms on clustering performance. In the dual-channel setting, removing either the node masking mechanism (Dual Ch, W/O Node M) or the edge masking mechanism (Dual Ch, W/O Edge M) resulted in ARI and ACC metrics that were superior to the Dual Ch, W/O M variant, which lacks both mechanisms. From the perspective of the identified spatial domains (Fig 7B), the Dual Ch, W/O Node M variant, which retains edge masking but lacks node masking, showed better clustering performance than the Dual Ch, W/O Edge M variant, which retains node masking but lacks edge masking. However, the spatial domains identified by Dual Ch, W/O Node M, such as Layer_2 and Layer_4, were thicker than those in the original cortical annotations, while Layer_3 was thinner. In contrast, although the clustering accuracy of Dual Ch, W/O Edge M was slightly lower, its identified cortical layer thickness was closer to manual annotations.

**Table 1:** Ablation studies on multiple datasets from various platforms to validate the significance of components of SpaMask contributions.

Method	Stereo-seq			osmFISH			MERFISH			ST		
	ARI	ACC	DIS	ARI	ACC	DIS	ARI	ACC	DIS	ARI	ACC	DIS
SpaMask	**0.748**	**0.755**	0.062	0.592	0.683	0.042	**0.523**	0.613	0.040	**0.560**	0.501	0.103
One Ch, Combine	0.747	0.751	0.064	**0.593**	0.684	0.038	0.521	**0.624**	0.033	0.541	0.485	0.108
Dual Ch, W/O M	0.518	0.531	0.116	0.553	0.670	0.037	0.407	0.507	0.060	0.421	0.424	**0.092**
Dual Ch, W/O Node M	0.743	0.752	**0.060**	0.589	0.682	0.033	0.515	0.620	0.034	0.522	0.488	0.102
Dual Ch, W/O Edge M	0.571	0.576	0.105	0.570	**0.698**	**0.028**	0.433	0.534	0.055	0.458	0.474	0.107
MGAE Ch, W/ M	0.268	0.392	0.175	0.381	0.548	0.065	0.250	0.431	0.048	0.545	0.500	0.120
MGAE Ch, W/O M	0.056	0.108	0.369	0.369	0.541	0.068	0.143	0.307	0.077	0.427	0.464	0.121
MGCL Ch, W/ M	0.724	0.742	0.061	0.544	0.683	0.038	0.513	0.610	**0.030**	0.435	0.430	0.100
MGCL Ch, W/O M	0.464	0.493	0.108	0.543	0.679	0.032	0.384	0.490	0.052	0.382	0.386	0.118
Pos Sel, Remain	0.651	0.650	0.090	0.579	0.668	0.036	0.489	0.576	0.040	0.554	**0.526**	0.098
Neg Sel, Random	0.744	0.753	0.063	0.586	0.683	0.036	0.508	0.600	0.031	0.511	0.497	0.109

To further validate the roles of the two masking mechanisms, we analyzed their performance in a single-channel setting to isolate the influence of the other channel. Masked variants of MGAE and MGCL outperformed their unmasked counterparts across multiple datasets. The MGAE Ch, W/O M variant showed the poorest spatial continuity on the Donor3 dataset, with an average dispersion (DIS) exceeding 0.055. Many spots were observed to mix along the boundaries of identified spatial domains, lacking clear separations. On the other hand, the MGCL channel demonstrated good continuity across all platforms due to its emphasis on node relationships, which prevents isolated nodes. However, MGCL tended to overestimate cortical thickness in Layers 2 and 4, while MGAE, despite exhibiting greater dispersion, provided a more consistent match with actual cortical thickness measurements. These results further validated the importance of the two masking mechanisms (Fig 7B). Finally, to more comprehensively evaluate the robustness of the method, we independently ran the experiments under 50 different random seeds and reported the mean (*μ*) and standard deviation (*σ*) of the metrics (S3 Table and S14 Fig).

From the above experiments, we conclude that the edge masking mechanism significantly enhances spatial clustering performance, while the node masking mechanism preserves the original cortical layer thickness as much as possible. Both mechanisms effectively reduce the occurrence of discrete, outlier spots. Under the shared-weight encoder framework, the two masking mechanisms mutually reinforce each other, achieving superior clustering performance while retaining the original structural features.

## 4. Discussion and conclusion

In this study, we introduce SpaMask for SRT analysis based on a dual masking mutual enhancement approach. SpaMask integrates Masked Graph Autoencoders (MGAE) and Masked Graph Contrastive Learning (MGCL) modules. The MGAE channel employs node masking to infer missing features based on spatial neighbor information, thus improving clustering accuracy. Meanwhile, the MGCL channel optimizes the embeddings of adjacent nodes through edge masking and contrastive loss, ensuring that spatially proximate nodes are more tightly clustered in feature space. Finally, a shared graph encoder is used to integrate the advantages of MGAE and MGCL.

We evaluated the the effectiveness of SpaMask on eight datasets from five different platforms. The experimental results demonstrate that SpaMask achieves greater consistency between identified spatial domains and structural layers across each dataset, with clearer organizational boundaries and fewer discrete spots within tissue structures. We comprehensively evaluated the spatial domain identification performance from multiple perspectives, showcasing SpaMask’s competitive capabilities and enhanced hierarchical continuity. Additionally, through denoising experiments, we explored the importance of denoised gene expression in identifying biologically relevant domains. When applied to multi-slice data analysis, SpaMask effectively integrated information across multiple contiguous slices, achieving the best batch correction FILISI score, indicating significant progress in eliminating batch effects.

We conducted ablation experiments to test the impact of SpaMask’s components on spatial domain clustering. These experiments revealed that both node masking and edge masking significantly enhance the clustering performance of spatial transcriptomics data. Compared to the no-masking scenario, node masking improved clustering accuracy while reducing dispersion, and edge masking achieved lower dispersion, preventing node isolation and further enhancing accuracy. These findings underscore the effectiveness of the dual masking strategy in optimizing spatial transcriptomics analysis, providing insights for future methodological advancements. In addition, we analyze and compare the computational performance of SpaMask, GraphST, STAGATE and SEDR on various datasets (See Sect J in S1 Text and S4 Table).

In summary, SpaMask offers an effective solution for analyzing spatial transcriptomics data, outperforming existing baseline methods in clustering performance, overcoming the limitations of traditional approaches, and effectively correcting batch effects in continuous slices.

## Supporting information

S1 TextSupplementary notes for SpaMask.(A) Key contributions of the paper. (B) Dataset description. (C) From frobenius norm to binary cross-entropy and graph contrastive learning. (D) Alignment of multiple consecutive slices. (E) Details on comparison with other spatial domain identification methods. (F) Evaluation citeria. (G) Selection of parameters *λ*, $\rho_{m}$ and $\rho_{d}$. (H) Clustering and UMAP details. (I) Detection of SVGs and spatially variable mete genes. (J) Computational cost.(PDF)

S1 Fig Multi-slice alignment process. (PDF)

S2 Fig The clustering accuracy of SpaMask with different hyperparameters in all 12 sections. (PDF)

S3 Fig The effect of applying PCA after latent representation on the clustering performance of SpaMask. (PDF)

S4 FigThe impact of different settings on SpaMask, including node masking rate $\rho_{m}$ , edge masking rate $\rho_{d}$ , and loss weight factor *λ.
*(PDF)

S5 Fig Comparison of spatial domains by clustering assignments via SpaMask and other methods in all 12 sections of the DLPFC dataset. (PDF)

S6 Fig UMAP visualization and PAGA graphs generated by SpaMask and other methods embeddings respectively. (PDF)

S7 Fig The SVGs and meta genes detected by SpaMask on the 151507 and 151673 slices. (PDF)

S8 Fig SpaMask_D performs denoising analysis on the 151507 slice. (PDF)

S9 Fig The spatial domain recognition results of SpaMask compared with other methods on human breast cancer, as well as the analysis of SpaMask_D on other clusters. (PDF)

S10 Fig The UMAP plots of SpaMask_D and other methods on human melanoma, as well as the denoising results of five specific genes by SpaMask_D. (PDF)

S11 Fig A further analysis was conducted on region 1 of the sagittal-anterior section of the mouse brain from the 10x Visium dataset, comparing the clustering performance of SpaMask with other methods on this dataset. (PDF)

S12 Fig SpaMask demonstrates improved multi-slice clustering performance on five slices of the mouse hypothalamic preoptic area. (PDF)

S13 Fig SpaMask achieves accurate horizontal integration across anterior and posterior datasets in mouse brains. (PDF)

S14 Fig The clustering accuracy of SpaMask in all 12 sections under the default hyperparameters with different random seeds. (PDF)

S1 Table Summary of the datasets in this study. (PDF)

S2 Table Summary of the clustering methods based on methodology, algorithm input and code link. (PDF)

S3 Table Mean (*μ*) and standard deviation (*σ*) of ARI and ACC metrics (expressed as  ( *μ* ± *σ* ) × 100) across four different platform datasets, evaluated using 50 independent experiments with random seeds for each method. (PDF)

S4 Table Comparative analysis of computational resource consumption, including Model Runtime (MR/Seconds), GPU Memory Usage (GMU/MB), and Memory Caching (MC/MB), across various models. (PDF)
