# Supplementary material for: SpaMask: Dual masking graph autoencoder with contrastive learning for spatial transcriptomics
Source: PLoS Comput Biol. 2025 Apr 3;21(4):e1012881. doi: 10.1371/journal.pcbi.1012881 (PMC11968113; doi:10.1371/journal.pcbi.1012881)
Supplement: S1 Text — (A) Key contributions of the paper. (B) Dataset description. (C) From frobenius norm to binary cross-entropy and graph contrastive learning. (D) Alignment of multiple consecutive slices. (E) Details on comparison with other spatial domain identification methods. (F) Evaluation citeria. (G) Selection of parameters λ, ρm and ρd. (H) Clustering and UMAP details. (I) Detection of SVGs and spatially variable mete genes. (J) Computational cost. (PDF) [file pcbi.1012881.s001.pdf]

Supplementary Materials for

SpaMask: Dual Masking Graph Autoencoder with Contrastive Learning for Spatial Transcriptomics

Supplementary Notes

A. Key contributions of the paper

- **Model Innovation**: This paper introduces SpaMask, a novel spatial transcriptomics analysis method that employs a dual-mask, mutually enhancing self-supervised learning framework. SpaMask integrates Masked Graph Autoencoder (MGAE) and Masked Graph Contrastive Learning (MGCL) techniques to generate robust latent representations of transcriptomic expression, significantly enhancing the accuracy of spatial domain clustering.
- **Methodological Contribution**: SpaMask incorporates two masking strategies, node masking and edge masking, allowing the model to infer masked node features using neighborhood information and to predict potential edge relationships from latent features. This dual-mask synergy, facilitated by a shared graph encoder, bolsters the model's robustness in handling spatial features, fully exploiting the intricate relationships between transcriptomic expression and spatial coordinates.
- **Experiments and Results**: Comprehensive evaluations on eight datasets across five platforms demonstrate that SpaMask excels in clustering accuracy, discreteness, and batch correction capabilities. Experimental results reveal that, compared with existing methods, SpaMask provides superior hierarchical continuity and clearer tissue boundaries in spatial domain recognition, successfully mitigates batch effects, and achieves the best FILISI score for batch correction in multi-slice data analysis.

B. Dataset description

We utilized eight datasets from five distinct platforms. First, from the 10x Visium platform, the datasets include the human dorsolateral prefrontal cortex (DLPFC), the human breast cancer (BRCA) dataset, and the anterior section 1 of mouse brain tissue (MBA) (1). The DLPFC dataset is provided by three independent donors, each offering four consecutive slices, totaling 12 slices. (2) manually annotated the DLPFC layers and white matter (WM) regions using morphological features and gene markers (`http://spatial.libd.org/spatialLIBD`). The BRCA dataset consists of 3,798 spots and 36,601 genes, with histological images exhibiting distinct regional shapes. This dataset, annotated by (3), covers 20 regions, including DCIS/LCIS, healthy tissue, invasive ductal carcinoma (IDC), and low-grade tumor margins (`https://support.10xgenomics.com/spatial-gene-expression/datasets/1.1.0/V1_Breast_Cancer_Block_A_Section_1`). The MBA dataset contains 2,695 spots and 32,285 genes. The Allen Mouse Brain Reference Atlas (4) has annotated this dataset with 51 known clusters and one unannotated area, totaling 52 identifiable spatial domains (`https://www.10xgenomics.com/datasets/mouse-brain-serial-section-1-sagittal-anterior-1-standard-1-0-0`) and the Allen Mouse Brain Reference Atlas (`https://mouse.brain-map.org/static/atlas`).

In addition, we included a human melanoma (HM) dataset from the ST platform, containing 293 spots and 16,148 genes (5). Melanoma, stroma, lymphoid tissue, and an unannotated region have been identified, creating four domains for spatial domain recognition evaluation (`https://www.spatialresearch.org/resources-published-datasets/doi-10-1158-0008-5472-can-18-0747/`).

With the rapid development of spatial transcriptomics technology, numerous platforms have emerged, making it essential to verify SpaMask's scalability and robust spatial domain recognition across datasets from diverse platforms. Thus, SpaMask was applied to high-resolution spatial transcriptomics datasets from the Stereo-seq platform, including the 9.5E mouse embryo and the mouse olfactory bulb. The mouse embryo dataset (6; 7) contains 5,913 spots and 23,015 genes, annotated into 12 regions, including AGM, Brain, Branchial arch, Cavity, Connective tissue, Dermomyotome, Heart, Liver, Mesenchyme, Neural crest, Notochord, and Sclerotome (`https://db.cngb.org/stomics/mosta/`). The mouse olfactory bulb dataset (8; 9) contains 10,000 spots and 26,145 genes, with annotations for eight regions: Subependymal Zone (SEZ), Mitral Layer (ML), Granule Cell Layer (GCL), Meninges, Olfactory Nerve Layer (ONL), External Plexiform Layer (OPL), Internal Plexiform Layer (IPL), and Glomerular Layer (GL) (`https://github.com/acheng416/Benchmark-CTCM-ST`).

We further applied SpaMask to a non-gridded dataset from the osmFISH platform (10; 11), representing the mouse somatosensory cortex (`http://linnarssonlab.org/osmFISH/availability`). This dataset includes 4,839 spots but only 33 genes, a significantly lower count than datasets from the 10x Visium and Stereo-seq platforms. The regions annotated include the Hippocampus, Internal Capsule Caudoputamen, lateral and medial Layer 2-3, Layers 3 to 6, Pia Layer 1, Ventricle, and White matter. Finally, SpaMask's spatial domain recognition was tested on five slices of the mouse hypothalamic preoptic area from the MERFISH platform (12), located at Bregma -0.04 mm, -0.09 mm, -0.14 mm, -0.19 mm and -0.24 mm. Each slice comprises eight domains and 155 genes. For further details, please refer to the table (`https://github.com/zhengli09/BASS-Analysis`).

C. From Frobenius Norm to Binary Cross-Entropy and Graph Contrastive Learning

Background

Consider a graph $\mathcal{G} = (\mathcal{V}, \mathcal{E})$, where $\mathcal{V}$ is the set of nodes and $\mathcal{E}$ is the set of edges. The adjacency matrix A is a $|\mathcal{V}| \times |\mathcal{V}|$ matrix, where $A_{uv} = 1$ if there is an edge between nodes u and v, and $A_{uv} = 0$ if there is no edge. The goal is to learn a latent representation $H \in \mathbb{R}^{|\mathcal{V}| \times d}$, where d is the dimensionality of the latent space, that can be used to reconstruct the graph structure.

From Frobenius Norm Loss to Binary Cross-Entropy Loss

Graph reconstruction is commonly approached as a binary classification problem, where the connectivity between node pairs is predicted as either an edge (1) or no edge (0). The Frobenius norm loss $\mathcal{L}_{\text{recon}}$ is typically used for this purpose, measuring the difference between the true adjacency matrix A and the reconstructed adjacency matrix $\hat{A}$:

$$\mathcal{L}_{\text{recon}} = \|\hat{A} - A\|_F^2 = \sum_{u,v} \left(\hat{A}_{uv} - A_{uv}\right)^2 \tag{1}$$

The decoder of the autoencoder produces $\hat{A} = H^\top H$, where $H^\top H$ represents the similarity matrix between nodes. This Frobenius norm loss minimizes the error between the actual and predicted adjacency matrix, optimizing H globally.

However, graph reconstruction can also be framed as a binary classification task, where each pair of nodes (u, v) is classified as connected or not. In this case, the binary cross-entropy (BCE) loss function is more appropriate. The predicted connection probability between nodes u and v is given by:

$$\Phi(h_u, h_v) = \sigma(h_u^\top h_v) \tag{2}$$

where σ is the sigmoid activation function:

$$\sigma(x) = \frac{1}{1 + e^{-x}} \tag{3}$$

The binary cross-entropy loss for each node pair is:

$$\mathcal{L}_{\text{BCE}} = -\sum_{u,v} \left[y_{uv} \log(\Phi(h_u, h_v)) + (1 - y_{uv}) \log(1 - \Phi(h_u, h_v))\right] \tag{4}$$

where y_{uv} is 1 if there is an edge between nodes u and v, and 0 otherwise. This loss directly measures the error between the predicted connection probabilities and the true labels, making it a natural extension of the Frobenius norm loss for graph reconstruction tasks.

The binary cross-entropy loss can be seen as a probabilistic interpretation of the Frobenius norm loss. By treating $h_u^\top h_v$ as the probability $\hat{A}_{uv}$ of a connection, the error $(h_u^\top h_v - A_{uv})$ from the Frobenius norm loss becomes a classification error. Therefore, the Frobenius norm loss approximates the binary cross-entropy loss when a sigmoid activation function is applied.

$$\mathcal{L}_{\text{recon}} \approx \mathcal{L}_{\text{BCE}} \tag{5}$$

From Binary Cross-Entropy Loss to Graph Contrastive Learning (GCL) Loss

While binary cross-entropy loss optimizes the connection prediction for each node pair, Graph Contrastive Learning (GCL) introduce an additional layer of sophistication by incorporating mutual information maximization. Mutual Information (MI) quantifies the dependence between two random variables. In the context of graph reconstruction, the goal is to maximize the similarity between latent representations of connected node pairs and minimize the similarity for non-connected pairs (13; 8).

Let the positive sample set $\mathcal{E}^+$ consist of the true edges in the graph, and the negative sample set $\mathcal{E}^-$ be generated through negative sampling, where node pairs without an edge are selected. For a node pair $(u, v) \in \mathcal{E}^+$, we want the predicted connection probability $\Phi(h_u, h_v)$ to be close to 1. For a node pair $(u', v') \in \mathcal{E}^-$, we want $\Phi(h_{u'}, h_{v'})$ to be close to 0. This leads to the following losses:

- Positive sample loss $\mathcal{L}^+$:

$$\mathcal{L}^+ = \frac{1}{|\mathcal{E}^+|} \sum_{(u,v)\in\mathcal{E}^+} \log \Phi(h_u, h_v) \tag{6}$$

- Negative sample loss $\mathcal{L}^-$:

$$\mathcal{L}^- = \frac{1}{|\mathcal{E}^-|} \sum_{(u',v')\in\mathcal{E}^-} \log(1 - \Phi(h_{u'}, h_{v'})) \tag{7}$$

The total loss function for Graph Contrastive Learning (GCL), $\mathcal{L}_{\text{GCL}}$, is then the negative sum of these positive and negative sample losses:

$$\mathcal{L}_{\text{GCL}} = -\left(\mathcal{L}^+ + \mathcal{L}^-\right) \tag{8}$$

Substituting the expressions for $\mathcal{L}^+$ and $\mathcal{L}^-$, we get:

$$\mathcal{L}_{\text{GCL}} = -\left(\frac{1}{|\mathcal{E}^+|} \sum_{(u,v)\in\mathcal{E}^+} \log \Phi(h_u, h_v) + \frac{1}{|\mathcal{E}^-|} \sum_{(u',v')\in\mathcal{E}^-} \log(1 - \Phi(h_{u'}, h_{v'}))\right) \tag{9}$$

This loss function optimizes the reconstruction of the graph by maximizing the similarity for positive samples (connected node pairs) and minimizing it for negative samples (non-connected node pairs).

The Frobenius norm loss $\mathcal{L}_{\text{recon}}$ optimizes the global error in reconstructing the adjacency matrix, while the binary cross-entropy loss $\mathcal{L}_{\text{BCE}}$ approaches graph reconstruction as a binary classification problem for each node pair. The binary cross-entropy loss can

be seen as a probabilistic version of the Frobenius norm loss, where it optimizes connection probabilities using a sigmoid activation function. Graph Contrastive Learning (GCL) extends this by incorporating mutual information maximization, which enables the model to effectively distinguish between positive and negative samples. The final Graph Contrastive Learning (GCL) function $\mathcal{L}_{\mathrm{GCL}}$ combines the positive and negative sample losses to optimize the overall graph structure reconstruction.

Modified Edge Noise Contrastive Loss

In the GCL loss described above, the similarity between node pairs (u, v) is computed as $\Phi(h_u, h_v) = \sigma(h_u^\top h_v)$ using the cosine similarity (dot product) of the latent representations. This implicitly assumes that the relationship between nodes is linear. However, node relationships are often more complex and cannot be represented adequately by a linear method. Therefore, we redefine Φ as a prediction module, Φ_ω, consisting of a feedforward neural network (FNN) with learnable parameters. We first compute the Hadamard product (element-wise product) of node pairs (u, v) and feed the resulting vector into a fully connected FNN to predict the similarity of node pair (u, v), which represents the connection probability between nodes u and v. The edge noise contrastive loss (NCE) (14) with learnable weights is then defined as follows:

$$\mathcal{L}_{\mathrm{NCE}} = -\left(\frac{1}{|\mathcal{E}^+|} \sum_{(u,v)\in\mathcal{E}^+} \log \Phi_\omega(h_u, h_v) + \frac{1}{|\mathcal{E}^-|} \sum_{(u',v')\in\mathcal{E}^-} \log(1 - \Phi_\omega(h_{u'}, h_{v'})) \right) \tag{10}$$

Here, $\Phi_\omega(h_u, h_v) = \sigma(\mathrm{FNN}(h_u \odot h_v))$. This approach provides greater flexibility to the model for learning complex node relationships, especially in scenarios involving high-order feature interactions. $\mathcal{E}^+$ denotes a set of positive edges, while $\mathcal{E}^-$ represents a set of negative edges sampled from the graph; typically, $\mathcal{E}^+ = \mathcal{E}$. However, in SpaMask, $\mathcal{E}^+$ is defined as the set of masked edges. $\mathcal{E}^-$ is obtained by randomly resampling a set of edges of the same size as $\mathcal{E}^+$.

D. Alignment of multiple consecutive slices

SpaMask extends single-slice data to continuous multi-slice data by aligning multiple slices through spatial point registration, facilitating batch correction using SpaMask. Prior to alignment, edge points in each slice are detected based on the number of neighboring points. For instance, in datasets acquired using the Visium platform, non-edge points are expected to have six neighboring points. For robustness, we define an edge point as one that has fewer than five neighboring points and more than one. Subsequently, SpaMask employs the Iterative Closest Point (ICP) algorithm to align edge points from two slices in pairs, assuming that edge points are typically located at the boundaries of tissues (15; 16). Let $P = \{p_1, p_2, \ldots, p_m\}$ represent the set of edge point locations in the source slice, and $Q = \{q_1, q_2, \ldots, q_k\}$ denote the set of edge point locations in the target slice, where $p_i, q_i \in \mathbb{R}^2$. The ICP algorithm aligns the points by iteratively executing the following steps until convergence:

1. For each point in the source point cloud P, find its closest point in the target point cloud Q to form a new set Q'. Then, $Q' = \{q'_1, q'_2, \ldots, q'_m\}$ containing points in the target point cloud is obtained.

2. Find the transformation with the optimal rotation and translation by solving the least squares problem.

$$\hat{R}, \hat{v} = \arg \min_{R\in\mathbb{R}^{2\times2}, v\in\mathbb{R}^2} \sum_{i=1}^{m} \|(Rp_i + v) - q'_i\|^2, \tag{11}$$

subject to $R^T R = I$.

3. Apply the transformation $\hat{R}p_i + \hat{v}$ to the source point cloud P.

For multiple slices, SpaMask performs pairwise alignment with the ICP algorithm sequentially.

E. Details on comparison with other spatial domain identification methods

In this study, we selected a range of representative state-of-the-art methods. For spatial domain identification in single slices, we chose two methods that leverage histological image features combined with spatial expression and coordinate information: SpaGCN and DeepST. Additionally, we included two reconstruction-based approaches: SEDR, which utilizes a deep autoencoder structure, and STAGATE, which employs an adaptive attention mechanism. We also incorporated two contrastive learning-based methods: CCST, based on the Deep Graph Infomax (DGI) approach, and GraphST, which uses DGI while also reconstructing the original features. Finally, we included DiffusionST, a method based on a diffusion model. For spatial domain identification across multiple slices, we adopted STAligner, Splane, STitch3D, and SPIRAL as baseline methods. The experimental setup is as follows:

- **SpaGCN** (17) integrates gene expression data, histological images, and spatial location information through Graph Convolutional Networks (GCNs) to identify variable genes within spatial domains. It employs unsupervised clustering algorithms to detect distinct spatial expression patterns. SpaGCN was applied to the entire dataset following the recommended parameters outlined in the package vignette.
- **DeepST** (18) is a spatial clustering method that utilizes graph autoencoders, taking gene expression data, histological images, and spatial information as inputs. It employs GCNs as encoders to reconstruct the input graph topology and identify features pertinent to spatial domain delineation. We retained the default parameters of DeepST, adjusting the parameter k to 12 for computing the adjacency matrix within the *get_graph* function. Additionally, because the human melanoma dataset lacks corresponding histological image data, we excluded histological information during testing.
- **CCST** (19) integrates spatial structure and gene expression information by stacking multiple layers of GCNs. It constructs positive and negative pairs using the maximum mutual information graph contrastive learning method to facilitate spatial transcriptomics analysis. CCST preprocessed the initial gene expression matrix using Principal Component Analysis (PCA) on

all spatial transcriptomics data, selecting the top 200 principal components as input dimensions. The selection of the radius ensured that each spot had between 5 and 10 neighbors. The overall CCST model utilized reference parameters provided in the original paper. Finally, the latent representations obtained were subjected to PCA to select the top 30 principal components, followed by the application of the mclust method to evaluate clustering performance.

- **SEDR** (3) employs a deep autoencoder network to learn gene representations while simultaneously embedding spatial information using a variational graph autoencoder. We executed SEDR for all experiments using the recommended parameters from the online tutorial (*run_SEDR_DLPFC_all_data.py*). Specifically, the parameter k was selected within the range of 6 to 10 based on clustering performance, and the number of epochs was set to 200.
- **STAGATE** (20) utilizes adaptive graph attention autoencoders to integrate spatial information with gene expression, thereby enhancing the accuracy of spatial domain identification. During preprocessing, STAGATE selected the top 3000 highly variable genes for each dataset and configured the radius to ensure that each spot had between 6 and 10 neighbors. All other parameters were consistent with those specified in the original publication.
- **GraphST** (21) employs a graph-based self-supervised contrastive learning approach that combines graph neural networks with self-supervised contrastive learning. By minimizing the embedding distance between spatially adjacent spots, it learns enriched and discriminative spot representations, and vice versa. Other parameters were set following the original paper's guidelines, with platform-specific models selected for datasets from platforms like Stereo and 10x Visium. After training, the latent representations for each spot are obtained, and the *refine_label()* function is used to optimize the clustering results. Additionally, GraphST provides processing methods for clustering across continuous multi-slice datasets, which we implemented consistently with the original paper's procedures.
- **DiffusionST** (22) utilizes a graph convolutional network model combined with a newly designed loss function and employs the zero-inflated negative binomial (ZINB) distribution for data denoising, followed by data augmentation using a diffusion model. DiffusionST is applied for spatial transcriptomics data imputation, with the imputed results validated by clustering performance. Initially, raw gene expression data undergoes filtering and normalization, with a threshold of 4,096 selected for feature genes. After obtaining the preprocessed gene expression count matrix, DenoiseST is used for feature learning, where a GCN model integrates each spot's features with those of its neighbors, and a ZINB model learns features for each gene to better fit the ZINB distribution. The data is then augmented using diffusion. Following clustering, the *refine_label()* function is also applied to optimize the clustering results.
- **STAligner** (23) utilizes STAGATE to learn latent representations and identifies MNN-pairs (mutual nearest neighbors) from this latent space. By applying a triplet loss function, it mitigates batch effects across multiple slices, enhancing multi-slice clustering performance. STAligner first integrates multiple slices and performs pretraining with STAGATE to learn the latent features of each spot. It then applies the MNN algorithm to construct positive and negative anchor pairs. Using triplet loss, it pulls similar anchor pairs closer across slices, thereby reducing batch effects between slices.
- **Splane** (24) uses Spoint to obtain the composition of labeled cell types from scRNA-seq data, which then serves as input for Splane. Spoint constructs a variational autoencoder (VAE) composed of encoder and decoder layers. Cells from the scRNA-seq dataset are used to simulate pseudo-spots, with each pseudo-spot's cell-type composition determined by its constituent cells. The deconvolution results from Spoint are subsequently input into Splane, which utilizes a combination of a GCN and adversarial learning to address batch effects across multiple slices. Splane operates in two stages: first, a discriminator is trained to develop discriminative capabilities, and second, features from different batches are mixed to mitigate batch effects across slices.
- **STitch3D** (16) maximizes overlap across multiple slices using ICP or PASTE algorithms, reconstructing gene expression from inferred cell compositions via scRNA-seq data. Input includes aligned slices with matching scRNA-seq, forming 3D spatial coordinates and a global neighborhood graph. STitch3D is then trained to integrate data across slices, creating a shared latent space that captures biological variations and corrects batch effects. In this latent space, each spot representation supports spatial domain recognition and cell-type deconvolution. STitch3D maps gene expression and spatial data into this shared space using a graph attention network. A discriminator further infers cell-type proportions from latent representations, enabling gene expression reconstruction by combining these proportions with cell-type-specific gene profiles.
- **SPIRAL** (25) integrates data from both feature space (including low-dimensional embeddings and high-dimensional gene expression) and physical space. It merges GraphSAGE and domain adaptation into a unified model to learn corrected embeddings and expression profiles by combining transcriptomic profiles with spatial context. GraphSAGE functions as the encoder, embedding gene expression and spatial coordinates into a low-dimensional latent space. A noise classifier and a biological discriminator are then designed to decompose these low-dimensional embeddings into two components: the noise component, used to identify batch-specific noise, and the biological component, used to integrate signals across batches. A decoder network reconstructs gene expression from these embeddings. The biological component of the low-dimensional embeddings is further grouped to reveal spatial domains across multiple samples.
- **stGCL** (26) is a multimodal model. It first uses a graph attention network (GAT) to extract features from gene expression data, resulting in gene expression embeddings. Then, a Vision Transformer (ViT) is employed to extract image feature information, which is further processed through the GAT to obtain image embeddings. The embeddings from the two modalities are integrated via contrastive learning, maximizing the mutual information between the gene embeddings and the image embeddings for each spot. When integrating multiple slices, stGCL requires coordinate alignment, which is divided into two types. For vertical multi-slice alignment, the provided `vertical_alignment` code is used to align the vertical slices. For horizontal slices, alignment is performed following the computational approach described in the paper. Finally, an adjacency matrix graph is constructed based on the integrated coordinates, which serves as input to the model.

F. Evaluation criteria

We utilize accuracy metrics to describe the clustering precision of this method, consistency metrics to assess the discreteness of the clustering results, and the local inverse Simpson index to evaluate batch correction effectiveness across multiple slices. Specifically, regarding the description of accuracy, we employ the Adjusted Rand Index (ARI) to compare the similarity between the clustering results and the manually annotated labels (27):

$$\mathrm{ARI} = \frac{\sum_{ij}\binom{N_{ij}}{2} - \frac{[\sum_i \binom{N_i}{2} + \sum_j \binom{N_j}{2}]}{\binom{N}{2}}}{\frac{1}{2}[\sum_i \binom{N_i}{2} + \sum_j \binom{N_j}{2}] - \frac{[\sum_i \binom{N_i}{2} + \sum_j \binom{N_j}{2}]}{\binom{N}{2}}} \tag{12}$$

where N is the total number of spots, N_{ij} represents the overlap of spots between the i-th predicted cluster ($C_i \in C$) and the j-th true cluster ($Y_j \in Y$), and N_i/N_j is the number of spots in cluster C_i/Y_j.

In addition, we have the Normalized Mutual Information (NMI) metric (28), which is based on information theory and measures the normalized mutual information between the clustering results and the true labels. The Homogeneity (HOM) score evaluates whether all clusters contain only data points belonging to a single class, indicating homogeneous clustering. The Completeness (COM) score assesses whether all data points belonging to a specific class are grouped within the same cluster, indicating complete clustering (29). The NMI is calculated as follows:

$$\mathrm{NMI}(Y, C) = \frac{2 \times [H(Y) - H(Y|C)]}{H(Y) + H(C)} \tag{13}$$

where the function $H()$ is used for calculating the entropy:

$$H(X) = -\sum_i p(x_i) \log p(x_i) \tag{14}$$

The HOM score is a metric that quantifies the homogeneity of a cluster labeling when compared to a known ground truth. A clustering outcome is considered homogeneous if all of its clusters exclusively comprise data points belonging to a single class. The HOM score is expressed as a value between 0 and 1, with 1 representing a perfectly homogeneous labeling.

The COM score is a metric that evaluates the completeness of a cluster labeling with respect to a ground truth. A clustering result is considered complete if all data points that belong to a certain class are grouped into the same cluster. The COM score ranges from 0 to 1, with a value of 1 indicating a perfect and complete labeling.

Therefore, the overall accuracy score is calculated as follows:

$$\mathrm{ACC} = \frac{1}{3} \times (\mathrm{NMI} + \mathrm{HOM} + \mathrm{COM}) \tag{15}$$

The closer the ARI and ACC scores are to 1, the better the clustering precision.

We assess spatial continuity using the Spatial Chaos Score (CHAOS), where a lower CHAOS value indicates better identified spatial domain continuity. The Percentage of Anomalous Points (PAS) represents the randomness of points located outside the clustered spatial domains, with a lower PAS score indicating better detected spatial domain continuity (29). The CHAOS score is a metric that has been used to assess the performance of spatial continuity in the mass spectrometry imaging field and the spatial transcriptomics field. A lower CHAOS indicates a better continuity result of spatial domain identification. To apply CHAOS to quantify each spatial clustering method's performance, we first build a 1-nearest neighbor (NN) graph for each dataset. Specifically, each cell is connected to another cell that has the minimum Euclidean distance in physical space. With this 1-NN graph, suppose d_{ij} is the Euclidean distance between cell i and cell j in physical space, we compute w as follows:

$$w_{kij} = \begin{cases} d_{ij}, & \text{if } \mathrm{cell}_i \text{ and } \mathrm{cell}_j \text{ are connected in the 1-NN graph in cluster } k \\ 0, & \text{otherwise} \end{cases} \tag{16}$$

Suppose n_k is the number of cells in the k th spatial domain, n is the total number of cells in the dataset, and K is the number of unique spatial domains.

$$\mathrm{CHAOS} = \frac{\sum_{k=1}^{K} \sum_{ij}^{n_k} w_{kij}}{N} \tag{17}$$

The PAS score has been used in quantifying the spatial homogeneity of spatial domain identification algorithms in the field of spatial transcriptomics. A lower PAS score indicates a better continuity of detected spatial domains, which expects higher cell homogeneity within spatial domains. The PAS score is calculated as the percentage of cells with a spatial domain label that is different from at least six of its neighboring ten cells.

Therefore, a lower overall score for discreteness corresponds to better continuity. The calculation is as follows:

$$\mathrm{DIS} = \frac{1}{2} \times (\mathrm{CHAOS} + \mathrm{PAS}) \tag{18}$$

We evaluate the degree of separation between the same domain and different domains by using the Local Inverse Simpson Index (LISI) for each batch within a domain (LISI_batch) and the LISI across all data for the domain (LISI_domain). The F1 score of the LISI (F1LISI) is calculated as follows (30):

$$\mathrm{F1LISI} = \frac{2(1 - \mathrm{LISI\_domain}_{norm})(\mathrm{LISI\_batch}_{norm})}{1 - \mathrm{LISI\_domain}_{norm} + \mathrm{LISI\_batch}_{norm}} \tag{19}$$

A higher F1LISI score indicates superior batch correction.

Moran's I is a metric to quantify the degree of spatial autocorrelation in spatial statistics (31; 29). The value of Moran's I ranges from -1 to 1. A value close to 1 indicates a clear spatial gene expression pattern, a value close to 0 indicates a random spatial gene expression pattern, and a value close to -1 indicates a gene expression pattern that resembles a chessboard. In our case, Moran's I will be used to evaluate the spatial autocorrelation computed by each method.

For one gene, suppose x_i and x_j are the gene expression values of spot i and spot j, $\bar{x}$ is the average gene expression value of the gene, and N is the number of spots. Then Moran's I is computed as:

$$\text{Moran's } I = \frac{N}{W} \frac{\sum_{i=1}^{N} \sum_{j=1}^{N} w_{ij}(x_i - \bar{x})(x_j - \bar{x})}{\sum_{i=1}^{N} (x_i - \bar{x})^2} \tag{20}$$

where

$$w_{ij} = \begin{cases} 1, & \text{if } i \text{ and } j \text{ are spatial neighbors} \\ 0, & \text{else} \end{cases}$$

and

$$W = \sum_{i,j} w_{ij}.$$

Like Moran's I, Geary's C is also a metric to quantify the degree of spatial autocorrelation in spatial omics analysis. The difference is that Geary's C ranges from 0 to 2, while Moran's I ranges from -1 to 1. Following the same set of notations as Moran's I, Geary's C is computed as follows:

$$\text{Geary's } C = \frac{N}{2W} \frac{\sum_{i=1}^{N} \sum_{j=1}^{N} w_{ij}(x_i - x_j)^2}{\sum_{i=1}^{N} (x_i - \bar{x})^2} \tag{21}$$

G. Selection of parameters λ, ρ_m and ρ_d

As demonstrated by the ablation experiments in the main text, the components of SpaMask are effective and robust. We further investigated three core hyperparameters that influence the model's performance: node masking rate, edge masking rate, and the weight factor λ that controls the trade-off between the reconstruction loss of the MGAE and the contrastive loss of the MGCL. To fully illustrate how these parameters affect clustering performance, we conducted ablation experiments on datasets from different platforms, including the Donor3 dataset from the 10x Visium platform, the mouse somatosensory cortex dataset from the osmFISH platform, and the mouse hypothalamic preoptic area in the bregma-0.04 mm region dataset from the MERFISH platform.

The ablation experiment results show the impact of the three hyperparameters (node masking rate, edge masking rate, and the weight factor λ) on clustering performance across different datasets. Specifically, the experiment tested different hyperparameter values for their effect on three evaluation metrics (ARI, ACC, and DIS) across the three datasets.

1. Impact of Node Masking Rate (ρ_m): The Visium and osmFISH datasets demonstrated strong robustness to changes in node masking rate. As the node masking rate increased, the clustering performance of these two datasets remained stable, especially within the lower masking rate range ($\rho_m \leq 0.5$), with metrics such as ARI and ACC showing minimal fluctuations. This indicates that the node features in these datasets are robust enough that even when some node information is lost, the model can still perform clustering effectively. This further suggests that for these datasets, clustering performance is primarily dependent on the MGCL channel with edge masking.

However, the MERFISH dataset showed greater sensitivity to changes in the node masking rate. As the masking rate increased, especially when exceeding 0.5, there was a significant decline in clustering performance, with a sharp drop in metrics. This suggests that node features are crucial for clustering results in the MERFISH dataset, and excessive loss of node information compromises intra-cluster coherence, leading to a marked deterioration in clustering quality.

Overall, when the node masking rate $\rho_m = 0.3$, it ensures that the clustering performance across different datasets is optimized, with good ARI, ACC, and lower DIS. Hence, the default node masking rate in SpaMask is set to 0.3.

2. Impact of Edge Masking Rate (ρ_d): A significant difference was observed in edge masking rate impacts across datasets. Overall, when the edge masking rate ρ_d reached 0.6, the ARI and ACC metrics for all datasets stabilized, with minimal fluctuation. This indicates that, at this point, the contribution of the MGCL channel with edge masking to the model's performance becomes negligible, and the model's performance is entirely dependent on the MGAE channel with node masking.

When $\rho_d = 0.5$, the results indicated that the osmFISH and MERFISH datasets (which utilize multiple fluorescence in situ hybridization (FISH) techniques) achieved the best ARI and ACC scores, with minimal data dispersion. Meanwhile, for the Visium platform, which uses high-throughput RNA sequencing, the best ARI and ACC values were observed at $\rho_d = 0.4$. This is because osmFISH and MERFISH benefit from a higher resolution (typically 1–2 µm), enabling cell-level or even single-molecule spatial localization analysis, whereas 10x Visium is suited for tissue-level analysis at a lower resolution (around 55 µm). The resolution differences between the datasets result in distinct neighbor relationships, which in turn affect the edge masking rate for inferred relationships between masked nodes. Consequently, SpaMask defaults to a edge masking rate of 0.5 for high-resolution datasets (e.g., FISH), while setting it to 0.4 for datasets with lower resolution.

3. Impact of Weight Factor (λ): The variations in the weight factor λ produced consistent results across different platforms. Increasing λ from 0.3 to 0.7 resulted in significant improvements in ARI and ACC, despite little change in the DIS metric. However, beyond $\lambda = 0.7$, the performance started to decline. The relatively stable DIS value indicates that intra-domain coherence remains steady for the dataset, and $\lambda = 0.7$ yields optimal clustering performance with good intra-domain cohesion and lower spot dispersion. This suggests that enhancing the weight of the contrastive loss effectively improves the model. These findings align

with our previous discussion on the components' effects on clustering performance, where the MGCL channel with edge masking significantly outperforms the MGAE channel with node masking in both clustering accuracy and coherence.

Thus, in SpaMask's dual-channel masking mechanism, assigning a higher weight to the MGCL channel contributes to optimizing clustering performance while preserving the node feature information.

In conclusion, our analysis and the results of the ablation experiments provide a comprehensive understanding of how key hyperparameters affect the performance of SpaMask and offer insights into configuring the model for optimal results across different datasets and platforms.

H. Clustering and UMAP Details

In this study, we utilized the k-means clustering method to analyze the latent representation H in order to identify distinct population structures within the dataset. K-means clustering is an unsupervised learning algorithm commonly employed for high-dimensional data, designed to partition a dataset into m predefined spatial domains. Initially, we defined the parameters for clustering, with the latent representation H serving as the input data matrix, which encapsulates the representation of each sample in the feature space. A critical aspect of clustering is the selection of an appropriate m, which corresponds to the number of cluster centers and aims to reflect the intrinsic structure and distribution characteristics of the data.

For the computation of UMAP (Uniform Manifold Approximation and Projection) (32), we first constructed a neighbor graph using the *sc.pp.neighbors* function to capture the local structural relationships between cells. In this step, we set the number of neighbors to 12 and selected the first 30 principal components of the latent representation H for the computation. Subsequently, we applied the *sc.tl.umap* function for UMAP dimensionality reduction, facilitating the visualization of similarities and differences among spots. Following this, we conducted *sc.tl.paga* analysis to elucidate the potential relationships between each spatial domain (33). The results were ultimately visualized using the *sc.pl.paga_compare* function.

I. Detection of SVGs and spatially variable mete genes

We detected Spatially Variable Genes (SVGs) (34; 35; 36) and meta-genes using the detection program provided by SpaGCN (17). To identify SVGs, we first predefined a circular neighborhood with a radius that ensures each spot in the target domain has approximately 10 neighbors. All spots in the neighboring domains of the target domain are then grouped into adjacent sets. If the total number of spots in a set exceeds 50, the neighboring domain is classified as adjacent. After identifying adjacent domains, we conducted differential testing using the Wilcoxon rank-sum test to compare spots in the target domain with those in the neighboring domains. Genes with adjusted P-values < 0.05 were selected as SVGs. To ensure that only genes with substantial expression patterns were included in the target domain, we further required the following three criteria: (1) the fraction of spots expressing the gene in the target domain, referred to as infraction, must exceed 80%; (2) the ratio of the percentage of spots expressing the gene in the target domain to that in each adjacent structural domain, termed the in/out score ratio, must be greater than 1; and (3) the fold change in expression between the target domain and adjacent domains must be greater than 1.5.

For the formation of meta-genes, we initially lowered the threshold for SVG filtering by adjusting the minimum fold change criterion from 1.5 to 1.2, allowing for the identification of genes exhibiting weaker enrichment expression patterns in the target domain. When multiple weak SVGs were present, we randomly selected one as the base gene, denoted as $gene_0$. The objective was to aggregate the expression from other genes to enhance the spatial pattern of the target domain. We first calculated the mean expression level e_0 of $gene_0$ in the target domain. Next, we extracted structural domains from non-target points where the expression of $gene_0$ exceeded e_0 to form the control group. Differential analysis was subsequently performed using the Wilcoxon rank-sum test, comparing spots in the target domain with those in the control group. Genes exhibiting the lowest false discovery rate (FDR)-adjusted P-values and higher expression in the target domain were selected as $gene_{0+}$. Similarly, we conducted differential analysis comparing the control group with genes from the target domain, selecting genes with the lowest FDR-adjusted P-values and expression levels higher than $gene_{0-}$ in the control group.

The expression of the meta-gene is calculated as follows:

$$\log(meta\_gene_1) = \log(gene_0) + \log(gene_{0+}) - \log(gene_{0-}) + C_0 \tag{22}$$

where C_0 is a constant ensuring that $\log(meta\_gene_1)$ remains non-negative. The logarithmic transformation is employed to rescale the expression levels, facilitating comparability among different genes. Including negative genes can enhance the spatial expression patterns of domains lacking enriched positive marker genes. This algorithm can be iteratively applied to identify additional genes, forming updated meta-genes with clearer spatial patterns in the target domain. For the $(t+1)$-th iteration, the expression of the meta-gene is computed as follows:

$$\log(meta\_gene_{t+1}) = \log(meta\_gene_t) + \log(gene_{t+}) - \log(gene_{t-}) + C_t \tag{23}$$

In the $(t+1)$-th iteration, after incorporating $gene_{t+}$ and subtracting $gene_{t-}$, the new control group is selected based on $meta\_gene_{t+1}$. The size of this control group, defined as the number of points outside the target domain but exhibiting higher $meta\_gene_{t+1}$ expression than those in the target domain, should be smaller than that of the t-th control group. This ensures that $meta\_gene_{t+1}$ has a clearer spatial pattern than $meta\_gene_t$. Furthermore, it is anticipated that the average expression difference between the target group and the control group for $meta\_gene_{t+1}$ will exceed that of $meta\_gene_t$. Thus, at each iteration, we verify whether these two criteria are satisfied; if not, the search for additional genes is terminated.

J. Computational cost

In this section, we analyze and compare the computational performance of SpaMask, GraphST, STAGATE, and SEDR on various datasets, including the Donor3 dataset from the 10x Visium platform, the human melanoma dataset from the ST platform, the mouse somatosensory cortex dataset from the osmFISH platform, the mouse olfactory bulb dataset from the Stereo-seq platform, and the bregma-0.04 mm region dataset from the mouse hypothalamic preoptic area using the MERFISH platform. The primary metrics of focus include **Model Runtime (MR), GPU Memory Usage (GMU), and Memory Caching (MC)**. These metrics effectively reflect differences in resource utilization and processing efficiency among different methods, which is particularly important when handling large-scale data.

During the comparison, we ensured that all experiments were conducted under identical hardware and software conditions to minimize external influences. Runtime directly impacts the model's practicality, so we measured the runtime of each method across different datasets to assess computational efficiency. GPU memory usage is another critical metric for evaluating a model's resource demands. We monitored the maximum memory capacity required by each method during execution and recorded peak memory usage. Optimizing GPU memory utilization is especially crucial for devices with limited memory capacity. Therefore, our comparative results offer valuable insights for researchers choosing an appropriate model.

Memory caching efficiency reflects how different methods utilize and manage cache during execution. We paid particular attention to memory fragmentation and data transfer frequency to identify differences in memory management strategies. Through comparison, we found that certain methods excelled in memory usage and cache management, while others focused more on minimizing runtime. We discuss each method's advantages and disadvantages, as well as their applicability under different scenarios, to provide a basis for future model optimization research.

SpaMask demonstrates moderate runtime performance across most datasets. For example, on the 10x Visium dataset, SpaMask's runtime is 63.15 seconds, which is faster than GraphST's 77.8 seconds but slower than STAGATE and SEDR. On the osmFISH dataset, SpaMask leads with the fastest runtime of 5.34 seconds compared to all other methods. Although some methods, such as SEDR, may exhibit faster runtimes on specific datasets, SpaMask consistently maintains high stability and strong performance across a diverse range of datasets.

SpaMask exhibits relatively low GPU memory consumption across various datasets. On the Stereo-seq and MERFISH datasets, it outperforms GraphST and STAGATE in terms of reduced memory usage, indicating effective memory management optimizations. On the 10x Visium dataset, SpaMask uses 71.41 MB of GPU memory, which is significantly lower than GraphST's 2307 MB and STAGATE's 237.65 MB. This exceptional memory utilization efficiency makes SpaMask especially suitable for devices with limited memory capacity.

In terms of memory caching, SpaMask shows robust performance. On the osmFISH dataset, SpaMask consumes 222 MB of memory caching, outperforming most other methods. Furthermore, on the 10x Visium dataset, SpaMask's memory caching usage stands at 2668 MB, which is lower than that of GraphST and other competing methods. By optimizing memory management and data transfer, SpaMask minimizes memory fragmentation and reduces data transfer frequency, striking a favorable balance between resource utilization and operational efficiency.

Overall, SpaMask stands out due to its adaptability and resource efficiency across various scenarios, providing robust spatial clustering while maintaining practical computational demands. This makes it an accessible choice for researchers with different computational constraints. We appreciate your attention to this matter and are confident that our findings offer valuable guidance for selecting the most appropriate computational models and optimizing future implementations in spatial transcriptomics research.

References

1. Ortiz C, Navarro JF, et al. Molecular atlas of the adult mouse brain. Science Advances. 2020;6(26):eabb3446–eabb3458.
2. Maynard KR, Collado-Torres L, et al. Transcriptome-scale spatial gene expression in the human dorsolateral prefrontal cortex. Nature Neuroscience. 2021;24(3):425–436.
3. Xu H, Fu H, et al. Unsupervised spatially embedded deep representation of spatial transcriptomics. Genome Medicine. 2024;16(1):12.
4. Sunkin SM, Ng L, et al. Allen Brain Atlas: an integrated spatio-temporal portal for exploring the central nervous system. Nucleic Acids Research. 2012;41(D1):D996–D1008.
5. Thrane K, Eriksson H, et al. Spatially resolved transcriptomics enables dissection of genetic heterogeneity in stage III cutaneous malignant melanoma. Cancer Research. 2018;78(20):5970–5979.
6. Chen A, Liao S, et al. Spatiotemporal transcriptomic atlas of mouse organogenesis using DNA nanoball-patterned arrays. Cell. 2022;185(10):1777–1792.
7. Richardson L, Venkataraman S, et al. EMAGE mouse embryo spatial gene expression database: 2014 update. Nucleic Acids Research. 2014;42(D1):D835–D844.
8. Nie W, Yu Y, Wang X, Wang R, Li SC. Spatially Informed Graph Structure Learning Extracts Insights from Spatial Transcriptomics. Advanced Science. 2024; p. 2403572.
9. Chen A, Liao S, Cheng M, Ma K, Wu L, Lai Y, et al. Spatiotemporal transcriptomic atlas of mouse organogenesis using DNA nanoball-patterned arrays. Cell. 2022;185(10):1777–1792.
10. Wang X, Allen WE, Wright MA, Sylwestrak EL, Samusik N, Vesuna S, et al. Three-dimensional intact-tissue sequencing of single-cell transcriptional states. Science. 2018;361(6400):eaat5691.
11. Codeluppi S, Borm LE, Zeisel A, La Manno G, van Lunteren JA, Svensson CI, et al. Spatial organization of the somatosensory cortex revealed by osmFISH. Nature Methods. 2018;15(11):932–935.
12. Moffitt JR, Bambah-Mukku D, Eichhorn SW, Vaughn E, Shekhar K, Perez JD, et al. Molecular, spatial, and functional single-cell profiling of the hypothalamic preoptic region. Science. 2018;362(6416):eaau5324.
13. Li J, Wu R, et al. What's Behind the Mask: Understanding Masked Graph Modeling for Graph Autoencoders. In: the 29th ACM SIGKDD Conference on Knowledge Discovery and Data Mining; 2023. p. 1268–1279.
14. Gutmann M, Hyvärinen A. Noise-contrastive estimation: A new estimation principle for unnormalized statistical models. In: Proceedings of the thirteenth international conference on artificial intelligence and statistics. JMLR Workshop and Conference Proceedings; 2010. p. 297–304.
15. Arun KS, Huang TS, Blostein SD. Least-squares fitting of two 3-D point sets. IEEE Transactions on pattern analysis and machine intelligence. 1987;(5):698–700.
16. Wang G, Zhao J, Yan Y, Wang Y, Wu AR, Yang C. Construction of a 3D whole organism spatial atlas by joint modelling of multiple slices with deep neural networks. Nature Machine Intelligence. 2023;5(11):1200–1213.
17. Hu J, Li X, et al. SpaGCN: Integrating gene expression, spatial location and histology to identify spatial domains and spatially variable genes by graph convolutional network. Nature Methods. 2021;18(11):1342–1351.
18. Xu C, Jin X, et al. DeepST: identifying spatial domains in spatial transcriptomics by deep learning. Nucleic Acids Research. 2022;50(22):e131–e131.
19. Li J, Chen S, et al. Cell clustering for spatial transcriptomics data with graph neural networks. Nature Computational Science. 2022;2(6):399–408.
20. Dong K, Zhang S, et al. Deciphering spatial domains from spatially resolved transcriptomics with an adaptive graph attention auto-encoder. Nature Communications. 2022;13(1):1739–1750.
21. Long Y, Ang KS, Li M, Chong KLK, Sethi R, Zhong C, et al. Spatially informed clustering, integration, and deconvolution of spatial transcriptomics with GraphST. Nature Communications. 2023;14(1):1155–1174.
22. Cui Y, Cui Y, Wang R, Nakai K, Ye X, Sakurai T, et al. DiffusionST: A diffusion model-based framework for enhancing spatial transcriptomics data quality and identifying spatial domains. Available at SSRN 4894131. 2024;.
23. Zhou X, Dong K, Zhang S. Integrating spatial transcriptomics data across different conditions, technologies and developmental stages. Nature Computational Science. 2023;3(10):894–906.
24. Xu H, Wang S, Fang M, Luo S, Chen C, Wan S, et al. SPACEL: deep learning-based characterization of spatial transcriptome architectures. Nature Communications. 2023;14(1):7603.
25. Guo T, Yuan Z, Pan Y, Wang J, Chen F, Zhang MQ, et al. SPIRAL: integrating and aligning spatially resolved transcriptomics data across different experiments, conditions, and technologies. Genome Biology. 2023;24(1):241–267.
26. Yu N, Zhang D, Zhang W, Liu Z, Qiao X, Wang C, et al. stGCL: A versatile cross-modality fusion method based on multi-modal graph contrastive learning for spatial transcriptomics. bioRxiv. 2023; p. 2023–12.
27. Rand WM. Objective criteria for the evaluation of clustering methods. Journal of the American Statistical Association. 1971;66(336):846–850.
28. Amelio A, Pizzuti C. Correction for closeness: Adjusting normalized mutual information measure for clustering comparison. Computational Intelligence. 2017;33(3):579–601.
29. Yuan Z, Zhao F, et al. Benchmarking spatial clustering methods with spatially resolved transcriptomics data. Nature Methods. 2024; p. 1–11.
30. Tran HTN, Ang KS, et al. A benchmark of batch-effect correction methods for single-cell RNA sequencing data. Genome Biology. 2020;21(1):1–32.
31. Miller BF, Bambah-Mukku D, Dulac C, Zhuang X, Fan J. Characterizing spatial gene expression heterogeneity in spatially resolved single-cell transcriptomic data with nonuniform cellular densities. Genome Research. 2021;31(10):1843–1855.

32. McInnes L, Healy J, et al. UMAP: Uniform Manifold Approximation and Projection. Journal of Open Source Software. 2018;3(29):861–911. doi:10.21105/joss.00861.
33. Wolf FA, Hamey FK, et al. PAGA: graph abstraction reconciles clustering with trajectory inference through a topology preserving map of single cells. Genome Biology. 2019;20(1):1–9.
34. Sun S, Zhu J, Zhou X. Statistical analysis of spatial expression patterns for spatially resolved transcriptomic studies. Nature Methods. 2020;17(2):193–200.
35. Svensson V, Teichmann SA, Stegle O. SpatialDE: identification of spatially variable genes. Nature Methods. 2018;15(5):343–346.
36. Zhang C, Dong K, et al. STAMarker: determining spatial domain-specific variable genes with saliency maps in deep learning. Nucleic Acids Research. 2023;51(20):e103–e103.
