# Supplementary material for: SpaMask: Dual masking graph autoencoder with contrastive learning for spatial transcriptomics
Source: PLoS Comput Biol. 2025 Apr 3;21(4):e1012881. doi: 10.1371/journal.pcbi.1012881 (PMC11968113; doi:10.1371/journal.pcbi.1012881)
Supplement: S2 Fig — (PDF) [file pcbi.1012881.s003.pdf]

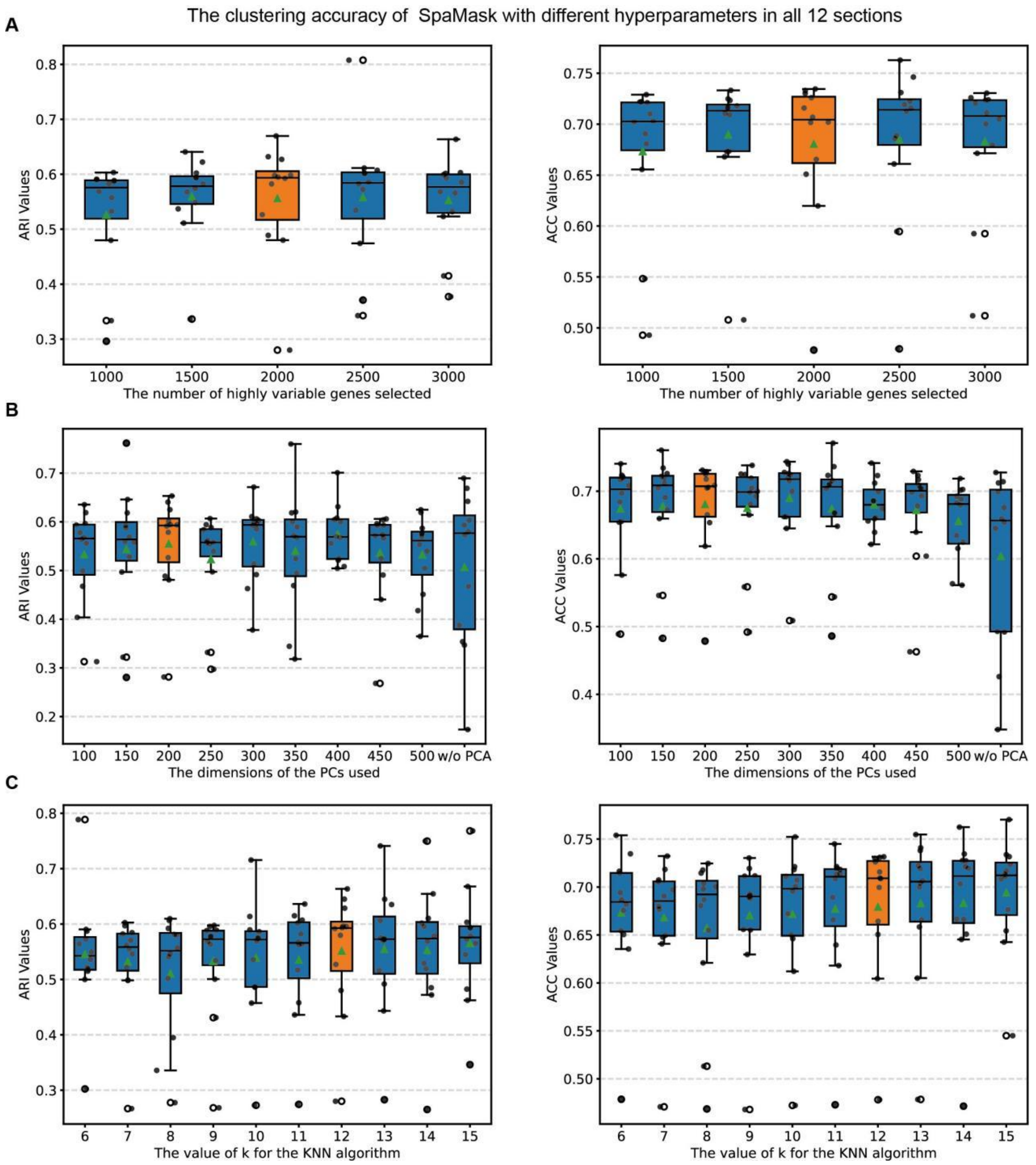

The clustering accuracy of SpaMask with different hyperparameters in all 12 sections. (A) Clustering performance (ARI and ACC boxplots) of SpaMask under different numbers of highly variable genes. **(B)** Clustering performance (ARI and ACC boxplots) of SpaMask under different numbers of principal components. **(C)** Clustering performance (ARI and ACC boxplots) of SpaMask under different values of K in the KNN algorithm for adjacency matrix construction.
