# Supplementary material for: SpaMask: Dual masking graph autoencoder with contrastive learning for spatial transcriptomics
Source: PLoS Comput Biol. 2025 Apr 3;21(4):e1012881. doi: 10.1371/journal.pcbi.1012881 (PMC11968113; doi:10.1371/journal.pcbi.1012881)
Supplement: S3 Fig — (PDF) [file pcbi.1012881.s004.pdf]

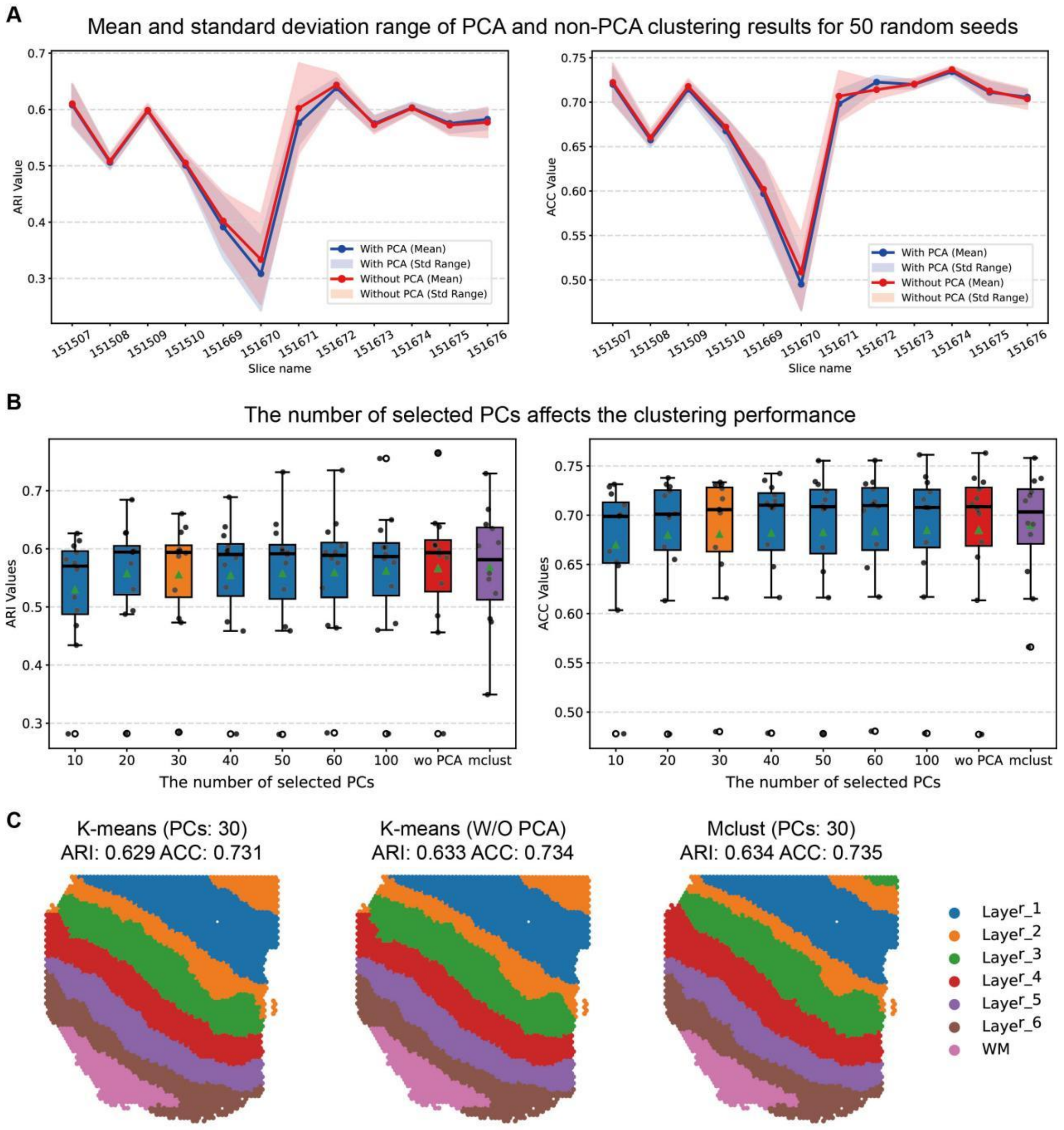

The effect of applying PCA after latent representation on the clustering performance of SpaMask. (A) Under the same parameter settings, ARI and ACC metrics were compared on the 12 slices of the DLPFC dataset using the PCA method versus without PCA. The results were evaluated across 50 different random seeds, recording the mean and variance of clustering outcomes for both methods. **(B)** The impact of varying the number of PCs on clustering performance. **(C)** Spatial domains identified using K-means clustering with 30 PCs, mclust clustering with 30 PCs, and K-means clustering without PCA directly on the embeddings.
