# Supplementary material for: SpaMask: Dual masking graph autoencoder with contrastive learning for spatial transcriptomics
Source: PLoS Comput Biol. 2025 Apr 3;21(4):e1012881. doi: 10.1371/journal.pcbi.1012881 (PMC11968113; doi:10.1371/journal.pcbi.1012881)
Supplement: S4 Fig — (PDF) [file pcbi.1012881.s005.pdf]

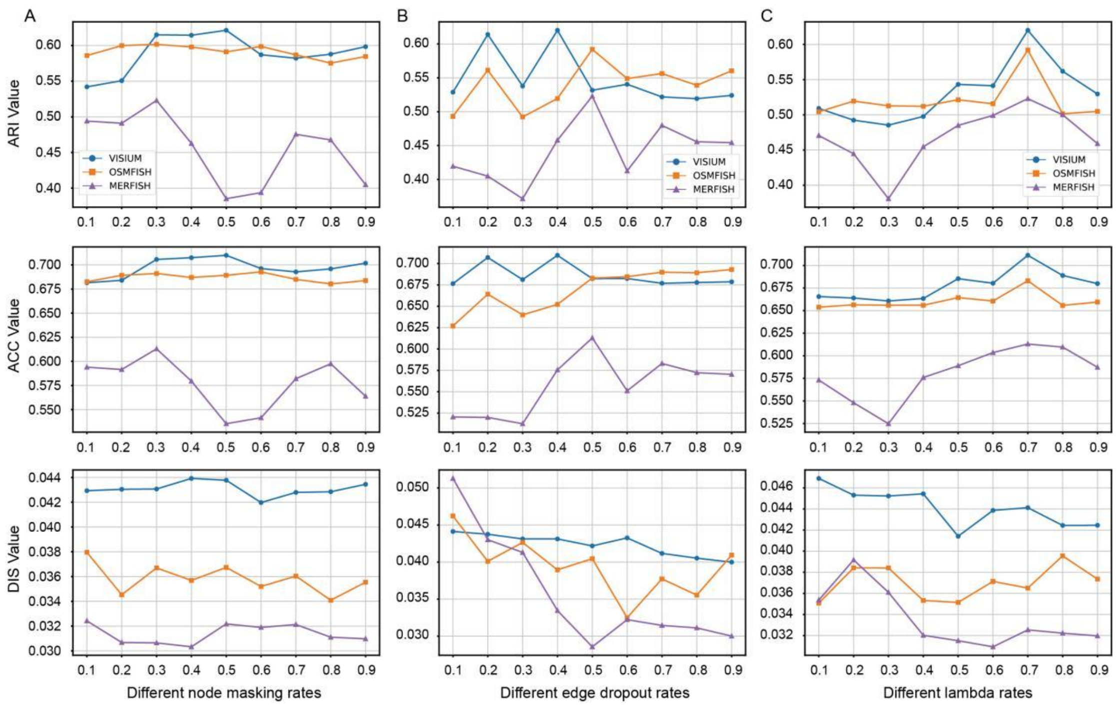

The impact of different settings on SpaMask, including node masking rate ρ_m, edge masking rate ρ_d, and loss weight factor λ. (A) The impact of varying node masking rates ρ_m. The three line charts in the upper, middle, and lower panels represent the effects of different node masking rates on the ARI, ACC, and DIS metrics across three different datasets. **(B)** The impact of varying edge masking rates ρ_d. Similarly, the three line charts in the upper, middle, and lower panels display the effects on ARI, ACC, and DIS for different datasets. **(C)** The impact of varying loss weight factor λ sizes. The three line charts in the upper, middle, and lower panels show the corresponding effects on the three metrics. Each of these sections provides insights into how different configuration parameters influence SpaMask's performance across multiple evaluation metrics.
