# Supplementary material for: SpaMask: Dual masking graph autoencoder with contrastive learning for spatial transcriptomics
Source: PLoS Comput Biol. 2025 Apr 3;21(4):e1012881. doi: 10.1371/journal.pcbi.1012881 (PMC11968113; doi:10.1371/journal.pcbi.1012881)
Supplement: S5 Fig — (PDF) [file pcbi.1012881.s006.pdf]

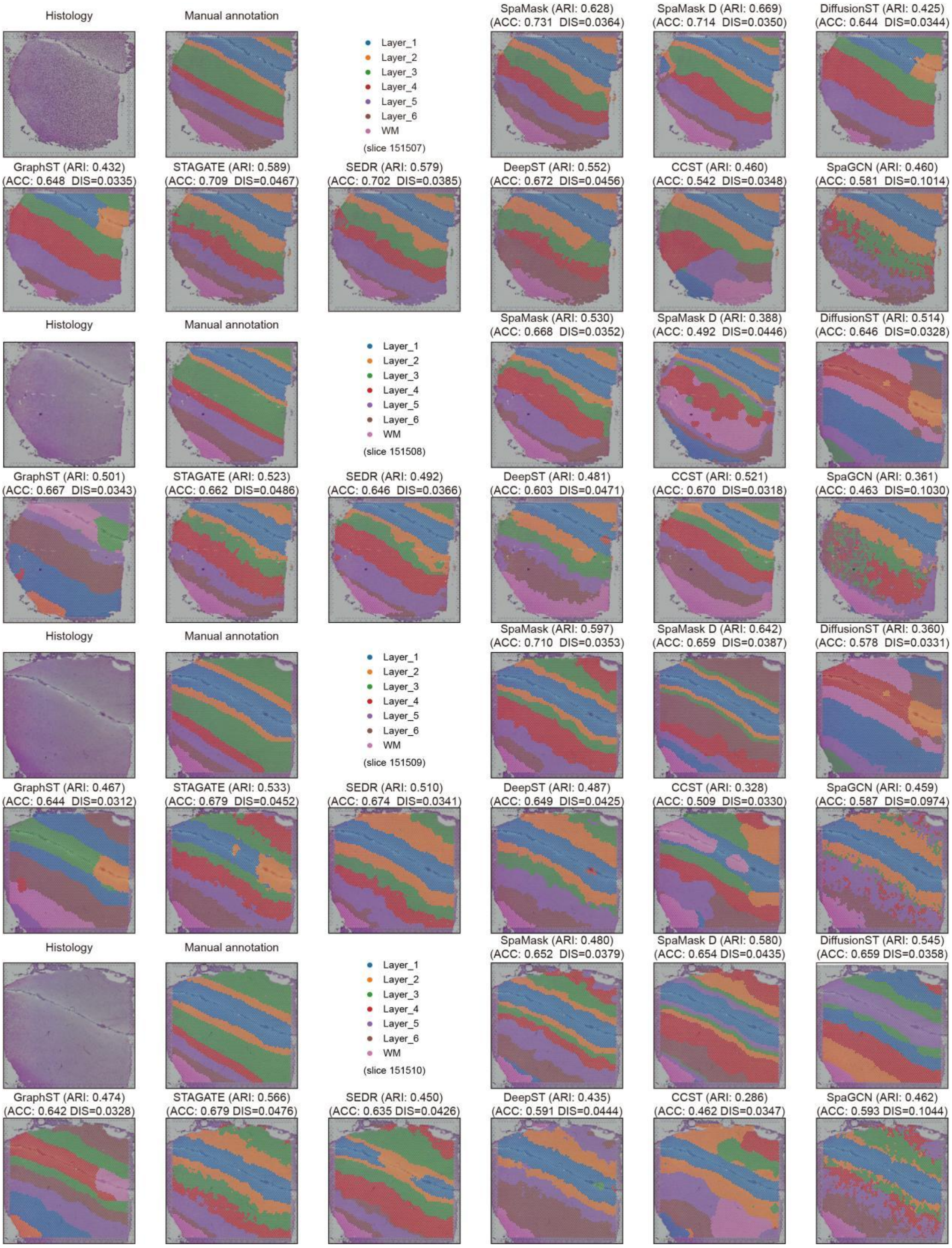

See next page

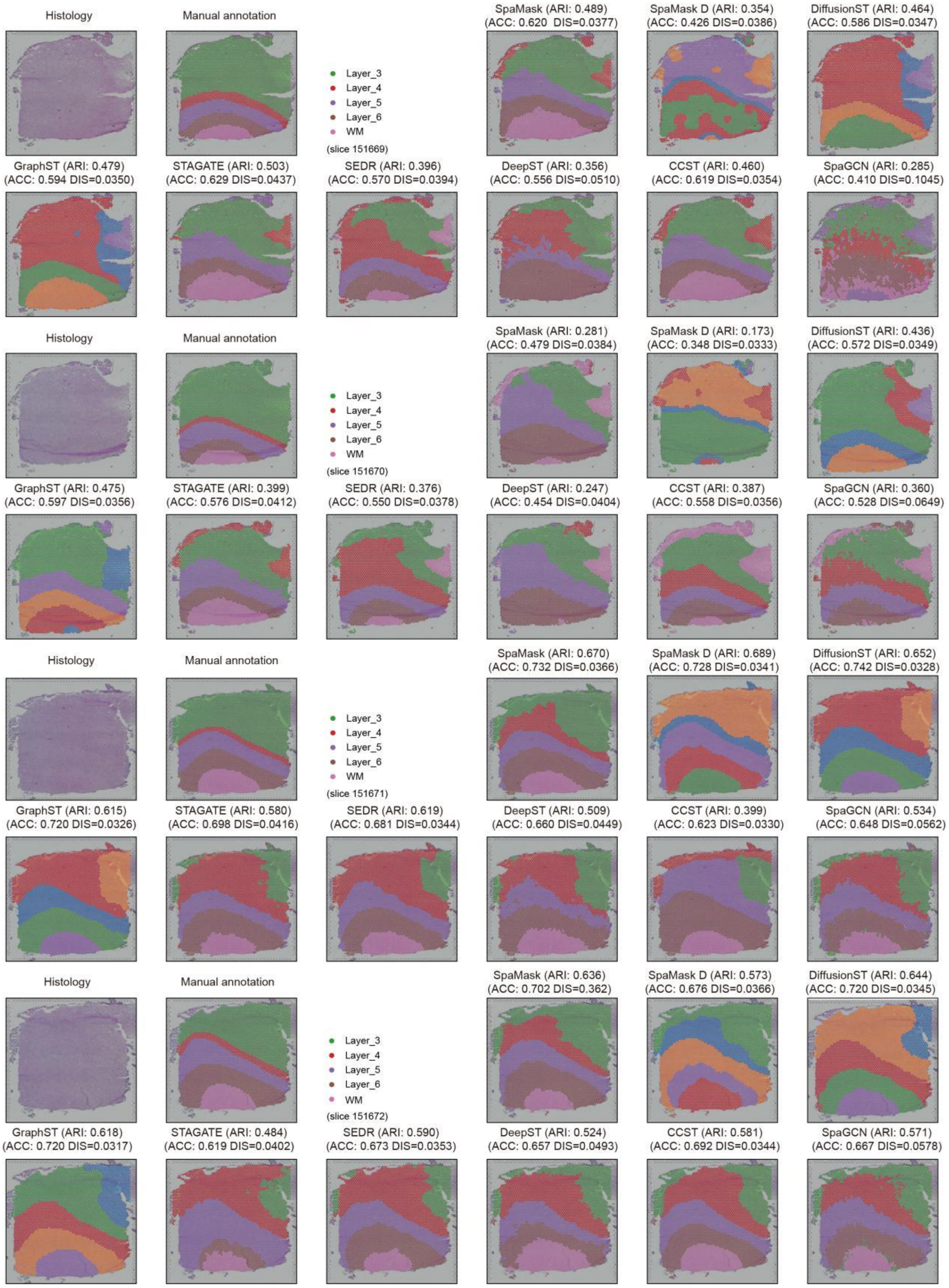

See next page

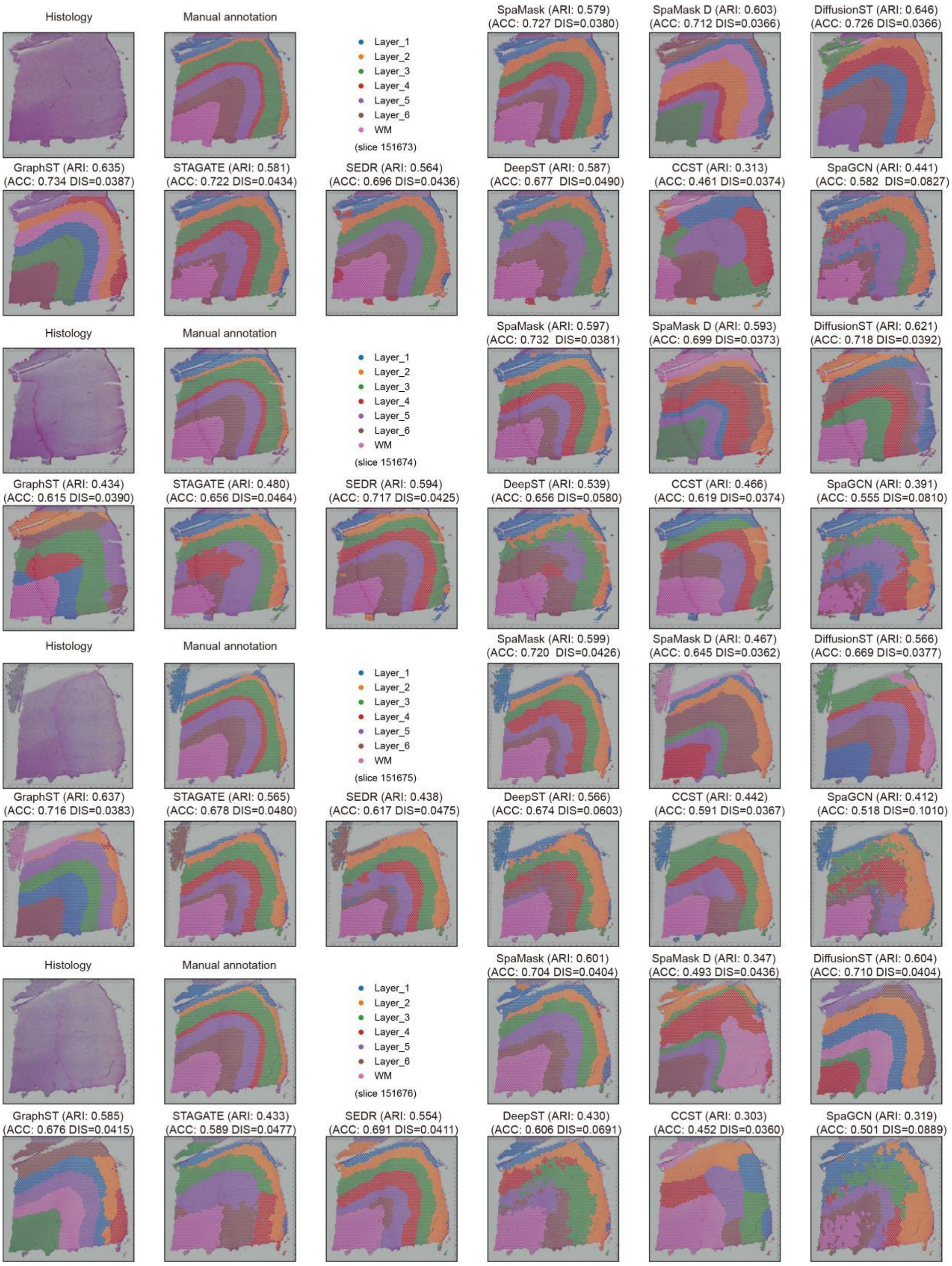

Comparison of spatial domains by clustering assignments via SpaMask, various methods, and manual annotation in all 12 sections of the DLPFC dataset.
