# Supplementary material for: SpaMask: Dual masking graph autoencoder with contrastive learning for spatial transcriptomics
Source: PLoS Comput Biol. 2025 Apr 3;21(4):e1012881. doi: 10.1371/journal.pcbi.1012881 (PMC11968113; doi:10.1371/journal.pcbi.1012881)
Supplement: S6 Fig — (PDF) [file pcbi.1012881.s007.pdf]

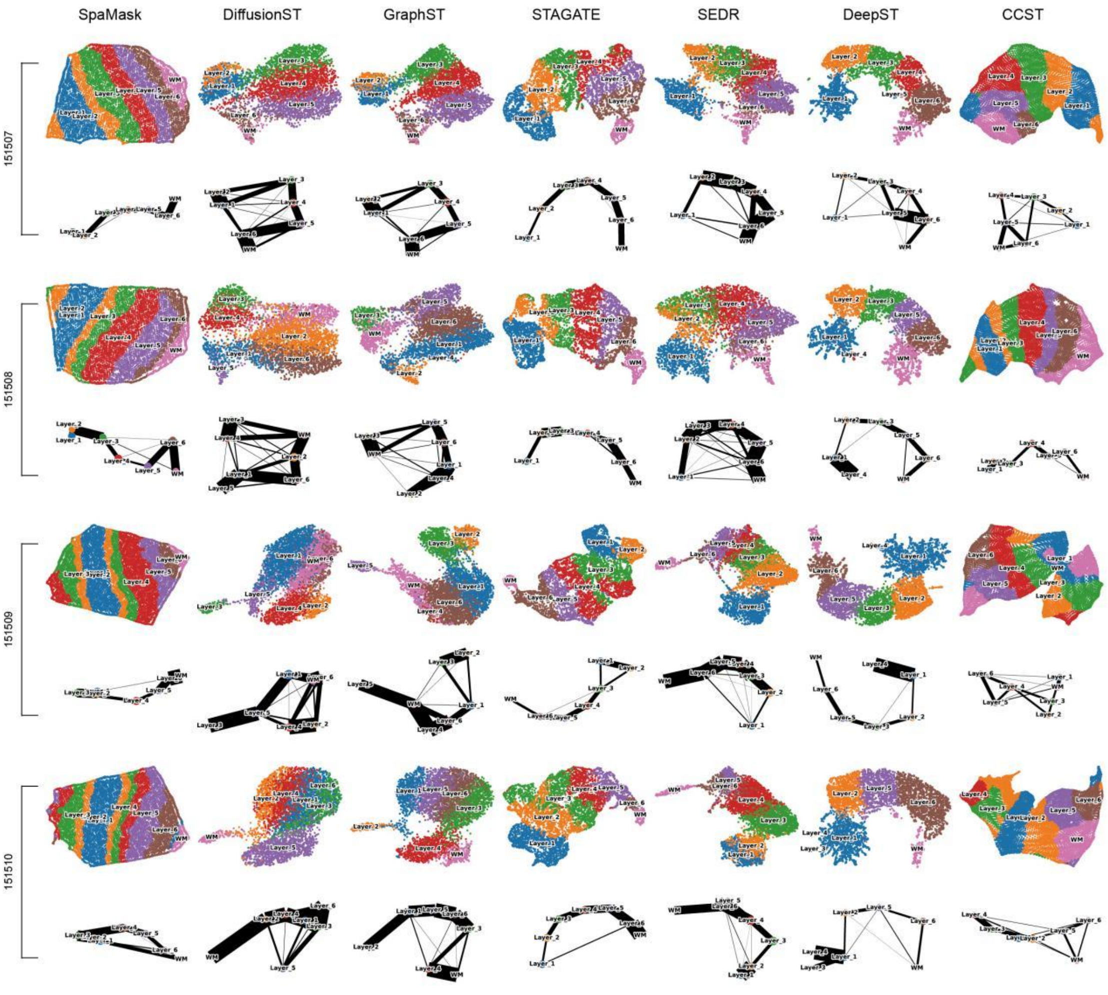

See next page

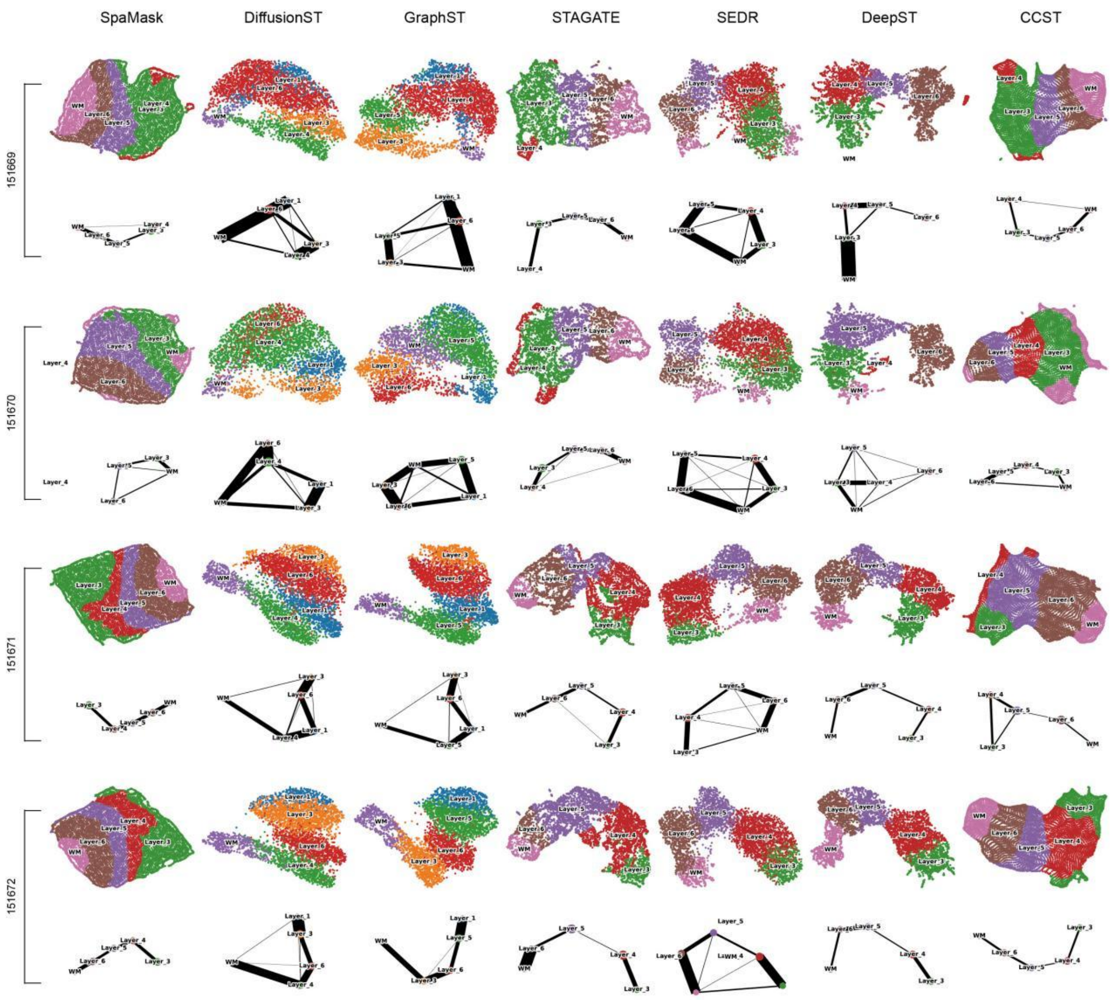

See next page

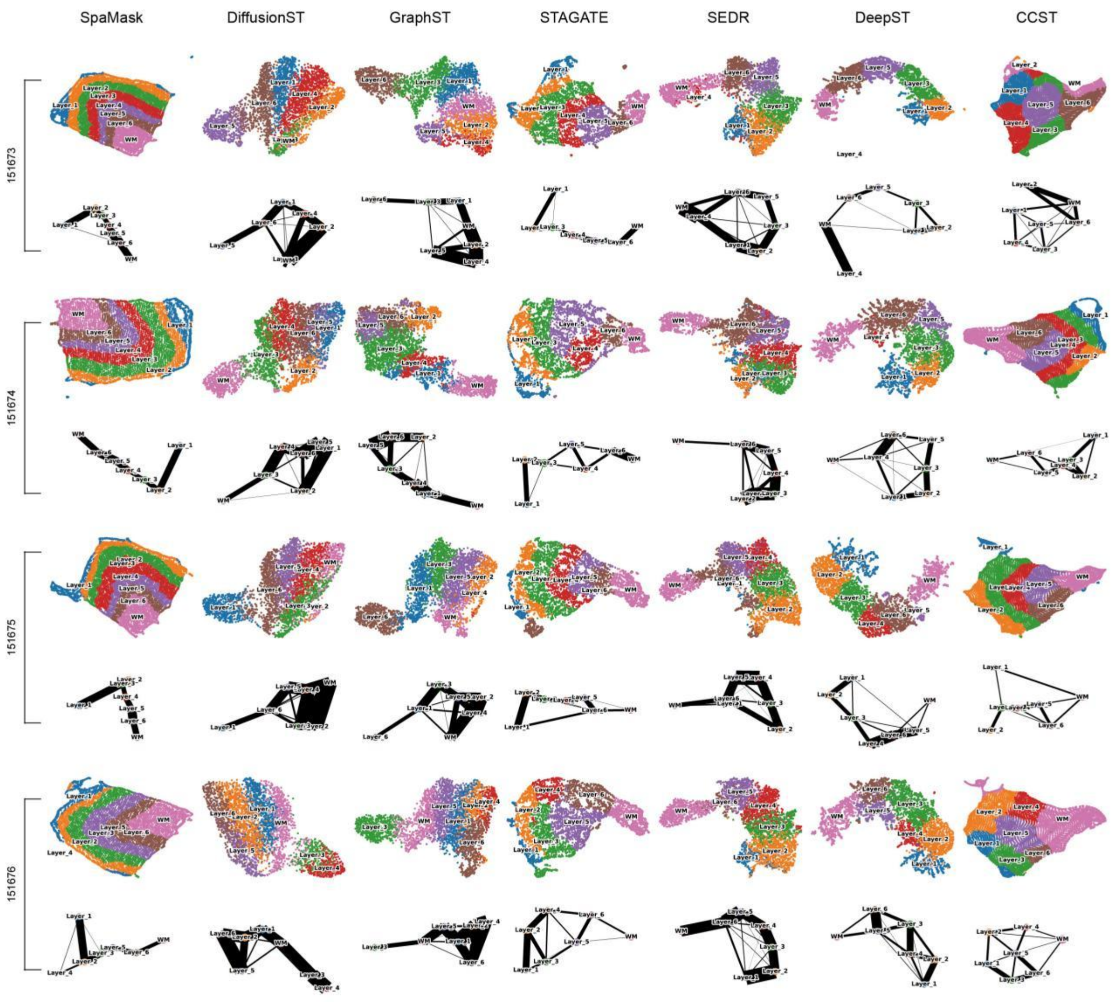

UMAP visualization and PAGA graphs generated by SpaMask and other various methods embeddings respectively.
