# Supplementary material for: SpaMask: Dual masking graph autoencoder with contrastive learning for spatial transcriptomics
Source: PLoS Comput Biol. 2025 Apr 3;21(4):e1012881. doi: 10.1371/journal.pcbi.1012881 (PMC11968113; doi:10.1371/journal.pcbi.1012881)
Supplement: S7 Fig — (PDF) [file pcbi.1012881.s008.pdf]

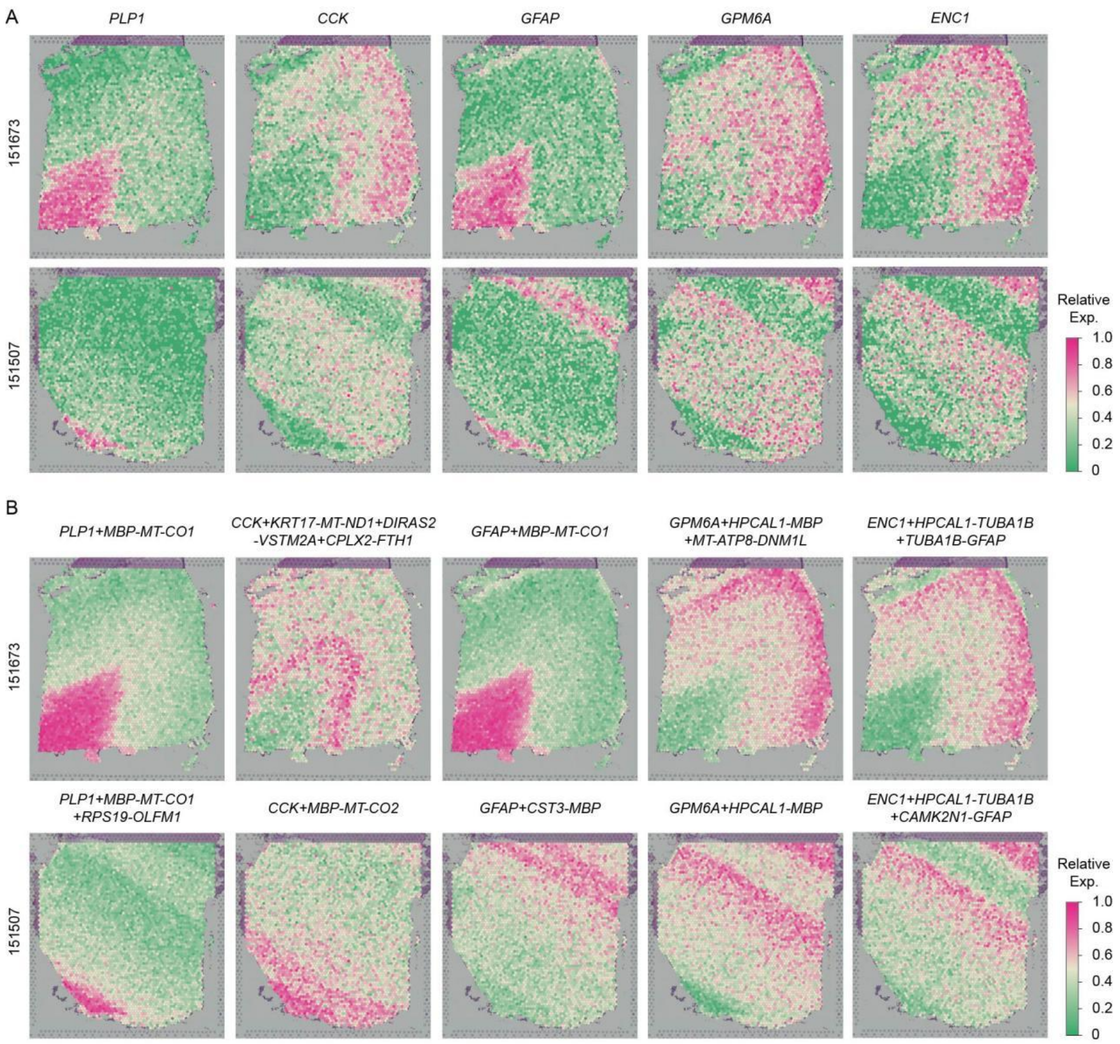

Detection of SVGs and spatially variable mete genes. (A) The five spatially variable genes detected by SpaMask on slices 151507 and 151673. **(B)** The spatially variable meta genes detected by SpaMask on slices 151507 and 151673.
