# Supplementary material for: SpaMask: Dual masking graph autoencoder with contrastive learning for spatial transcriptomics
Source: PLoS Comput Biol. 2025 Apr 3;21(4):e1012881. doi: 10.1371/journal.pcbi.1012881 (PMC11968113; doi:10.1371/journal.pcbi.1012881)
Supplement: S8 Fig — (PDF) [file pcbi.1012881.s009.pdf]

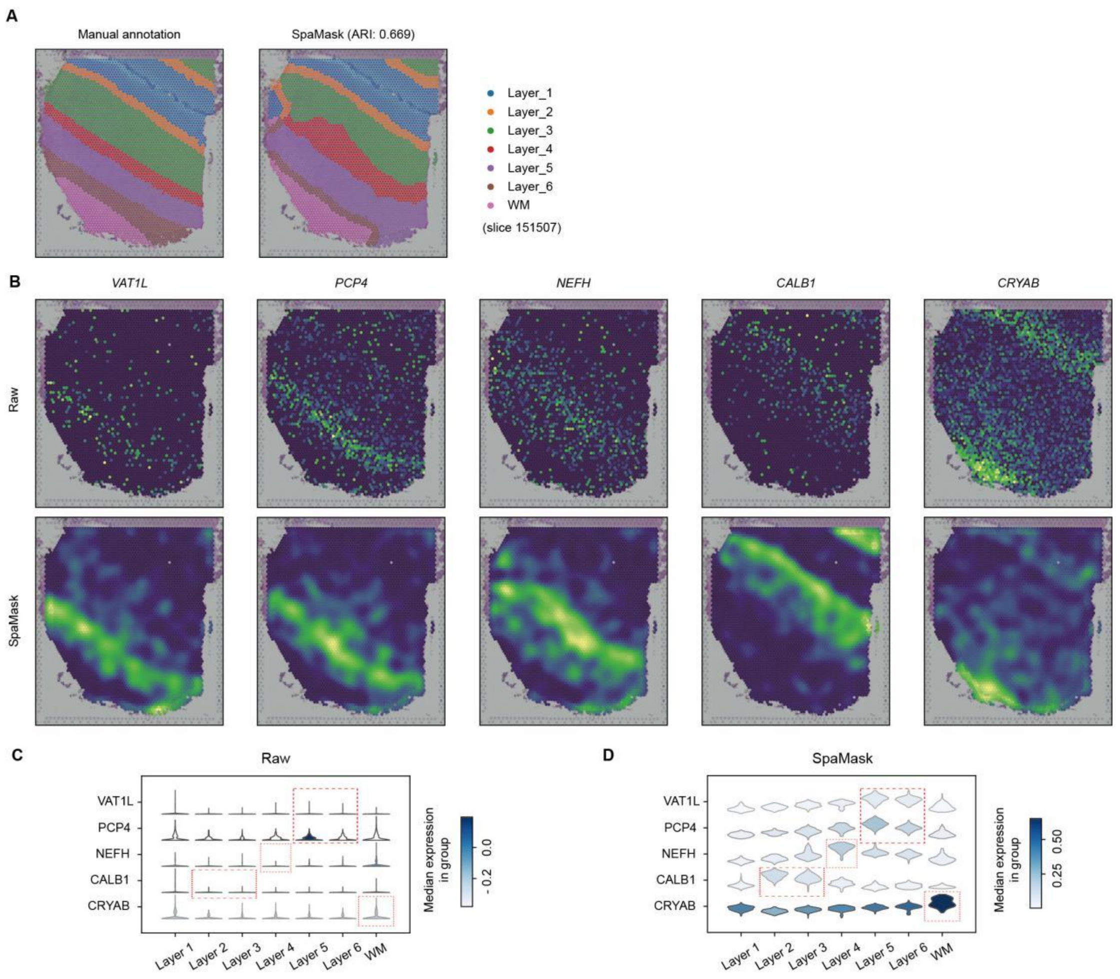

Comparison of spatial expression patterns before and after SpaMask denoising. (A) Ground-truth segmentation of cortical layers and white matter (WM) in the DLPFC section 151507. **(B)** Expression visualization of five layer-marker genes in the DLPFC section 151507. **(C)** and **(D)** Violin plots showing the raw expression **(C)** and SpaMask-denoised expression **(D)** of layer-marker genes in the DLPFC section 151507. The cortical layer corresponding to each layer-marker gene is highlighted with red boxes.
