# Supplementary material for: SpaMask: Dual masking graph autoencoder with contrastive learning for spatial transcriptomics
Source: PLoS Comput Biol. 2025 Apr 3;21(4):e1012881. doi: 10.1371/journal.pcbi.1012881 (PMC11968113; doi:10.1371/journal.pcbi.1012881)
Supplement: S9 Fig — (PDF) [file pcbi.1012881.s010.pdf]

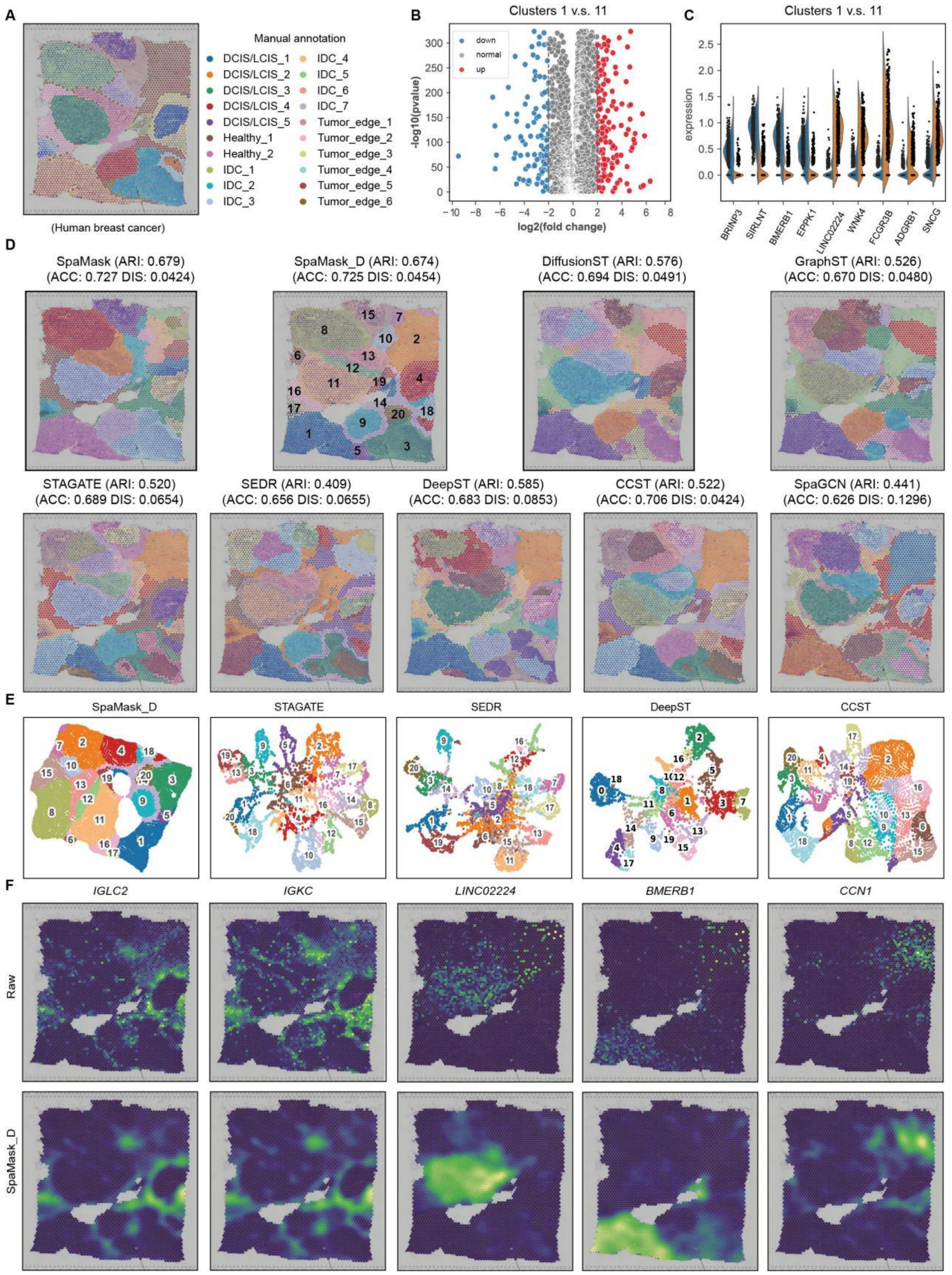

Experiment results in the human breast cancer dataset. (A) Manually annotated. **(B)** Volcano plot of DEGs between DCIS/LCIS (Cluster 11) region and IDC (Cluster 1) region. **(C)** Differential expression analysis between DCIS/LCIS (Cluster 11) region and IDC (Cluster 1) region. **(D)** Spatial domains detected by various methods. **(E)** UMAP visualization generated through embedding. **(F)** Expression visualization of five layer-marker genes in the human breast cancer dataset.
