# Supplementary material for: SpaMask: Dual masking graph autoencoder with contrastive learning for spatial transcriptomics
Source: PLoS Comput Biol. 2025 Apr 3;21(4):e1012881. doi: 10.1371/journal.pcbi.1012881 (PMC11968113; doi:10.1371/journal.pcbi.1012881)
Supplement: S10 Fig — (PDF) [file pcbi.1012881.s011.pdf]

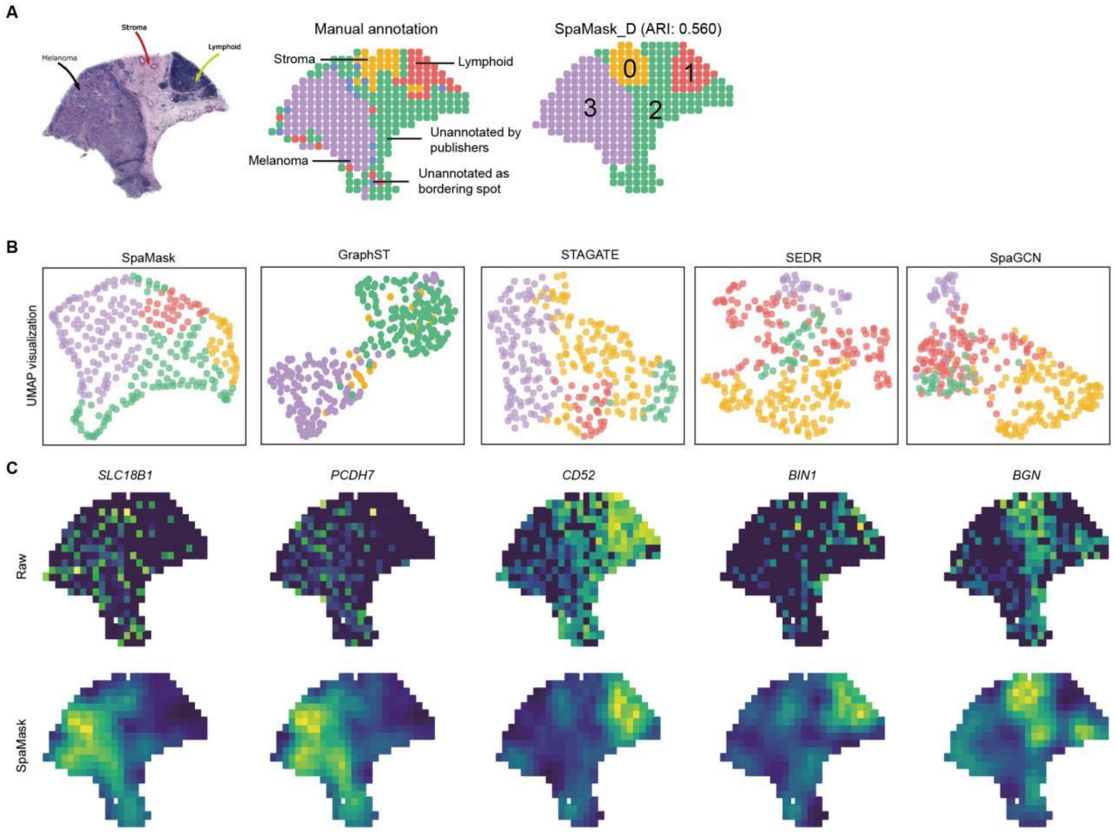

Experiment results in the human melanoma dataset. (A) Tissue images, manually annotated and spatial domains detected by SpaMask_D. **(B)** UMAP visualization generated through embedding. **(C)** Expression visualization of five layer-marker genes in the human melanoma dataset.
