# Supplementary material for: SpaMask: Dual masking graph autoencoder with contrastive learning for spatial transcriptomics
Source: PLoS Comput Biol. 2025 Apr 3;21(4):e1012881. doi: 10.1371/journal.pcbi.1012881 (PMC11968113; doi:10.1371/journal.pcbi.1012881)
Supplement: S11 Fig — (PDF) [file pcbi.1012881.s012.pdf]

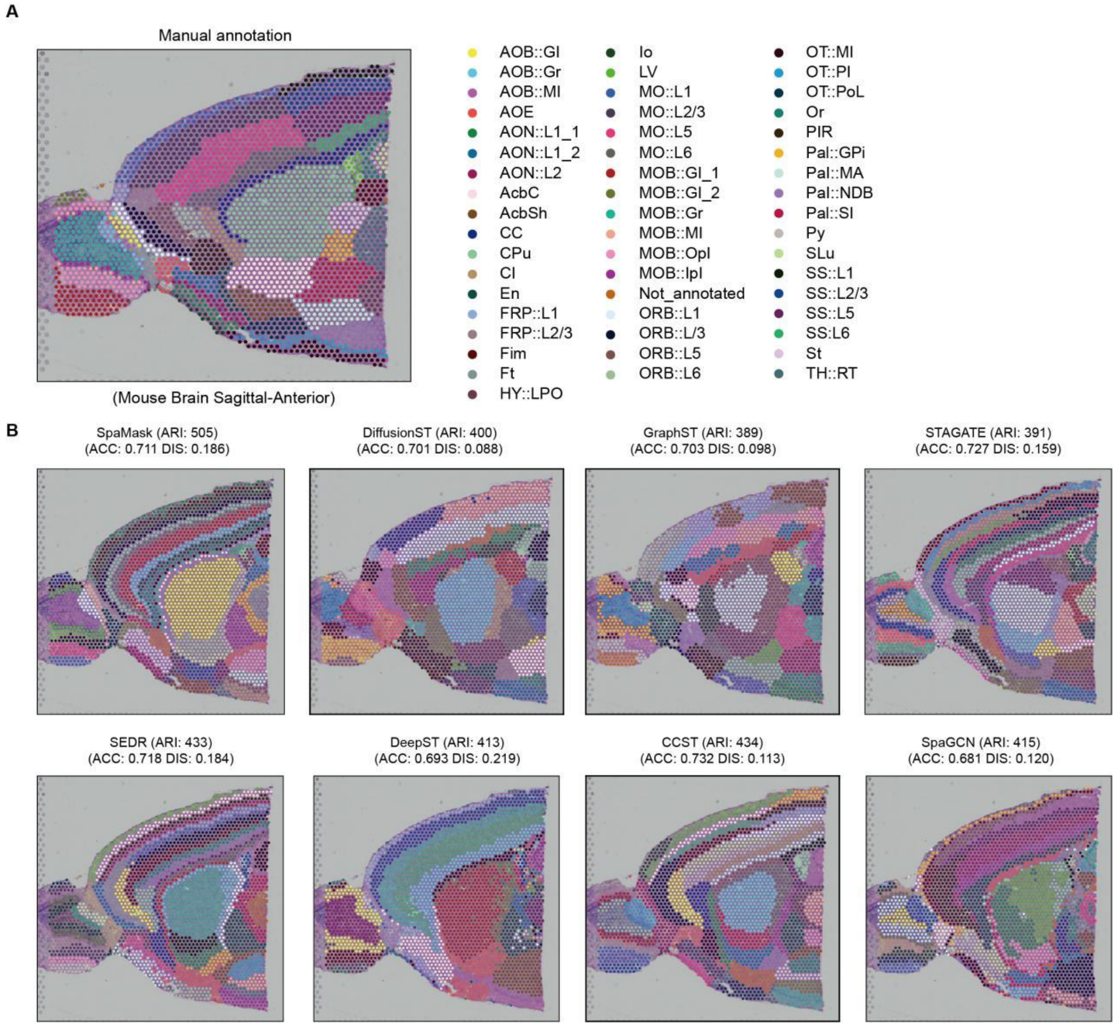

Experiment results in the mouse brain dataset. (A) Manually annotated. **(B)** Spatial domains detected by various methods on mouse brain.
