# Supplementary material for: SpaMask: Dual masking graph autoencoder with contrastive learning for spatial transcriptomics
Source: PLoS Comput Biol. 2025 Apr 3;21(4):e1012881. doi: 10.1371/journal.pcbi.1012881 (PMC11968113; doi:10.1371/journal.pcbi.1012881)
Supplement: S12 Fig — (PDF) [file pcbi.1012881.s013.pdf]

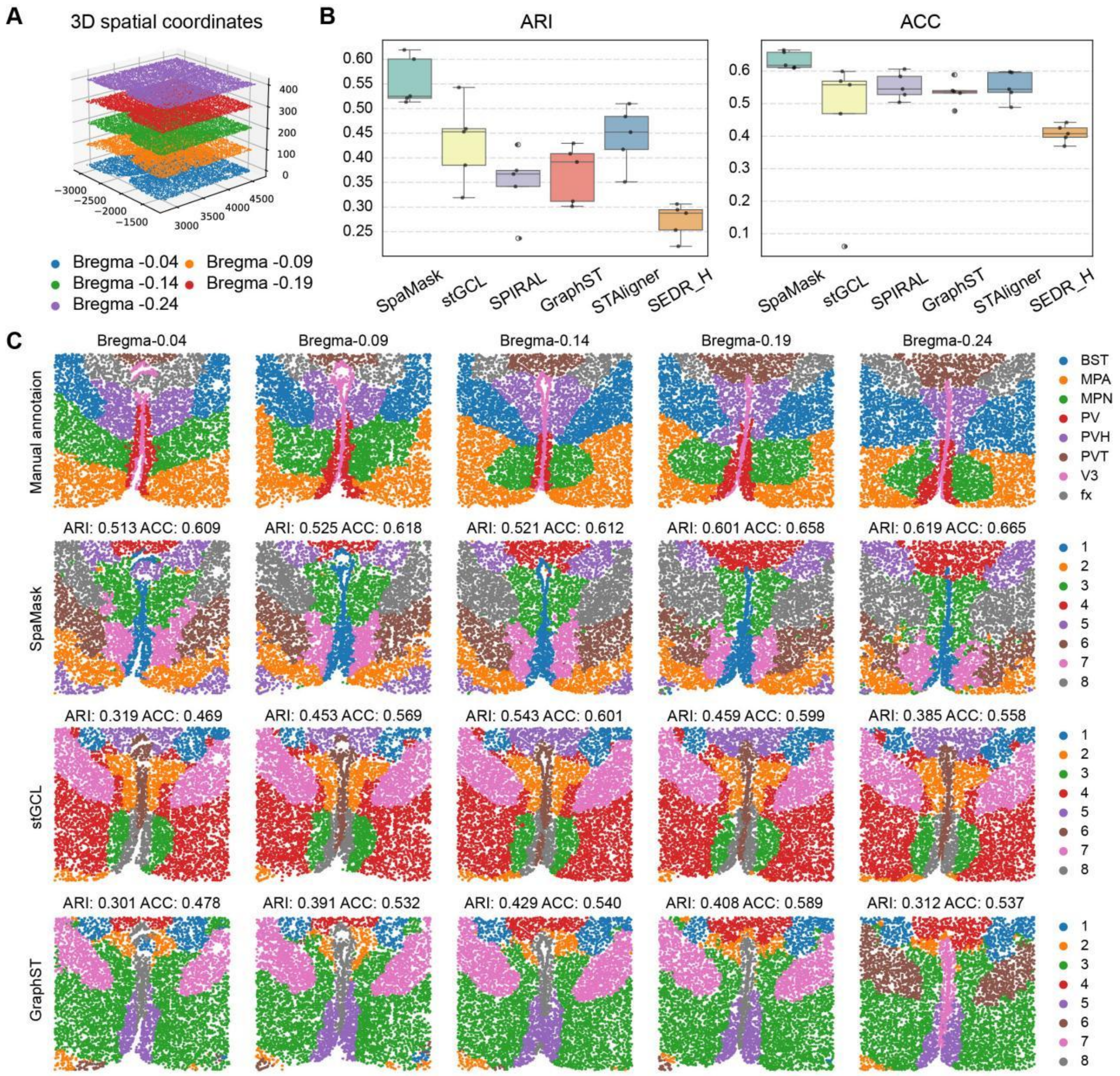

SpaMask demonstrates improved multi-slice clustering performance on five slices of the mouse hypothalamic preoptic area. (A) Slices located at Bregma-0.04 mm, -0.09 mm, -0.14 mm, -0.19 mm, and -0.24 mm regions were aligned to construct 3D spatial coordinates. **(B)** Box plots comparing SpaMask with various methods (stGCL, SPIRAL, GraphST, STAligner, SEDR_Harmony) across ARI and ACC metrics. **(C)** Spatial domains identified by SpaMask, stGCL, and GraphST, demonstrating the effectiveness of SpaMask in domain detection.
