# Supplementary material for: SpaMask: Dual masking graph autoencoder with contrastive learning for spatial transcriptomics
Source: PLoS Comput Biol. 2025 Apr 3;21(4):e1012881. doi: 10.1371/journal.pcbi.1012881 (PMC11968113; doi:10.1371/journal.pcbi.1012881)
Supplement: S13 Fig — (PDF) [file pcbi.1012881.s014.pdf]

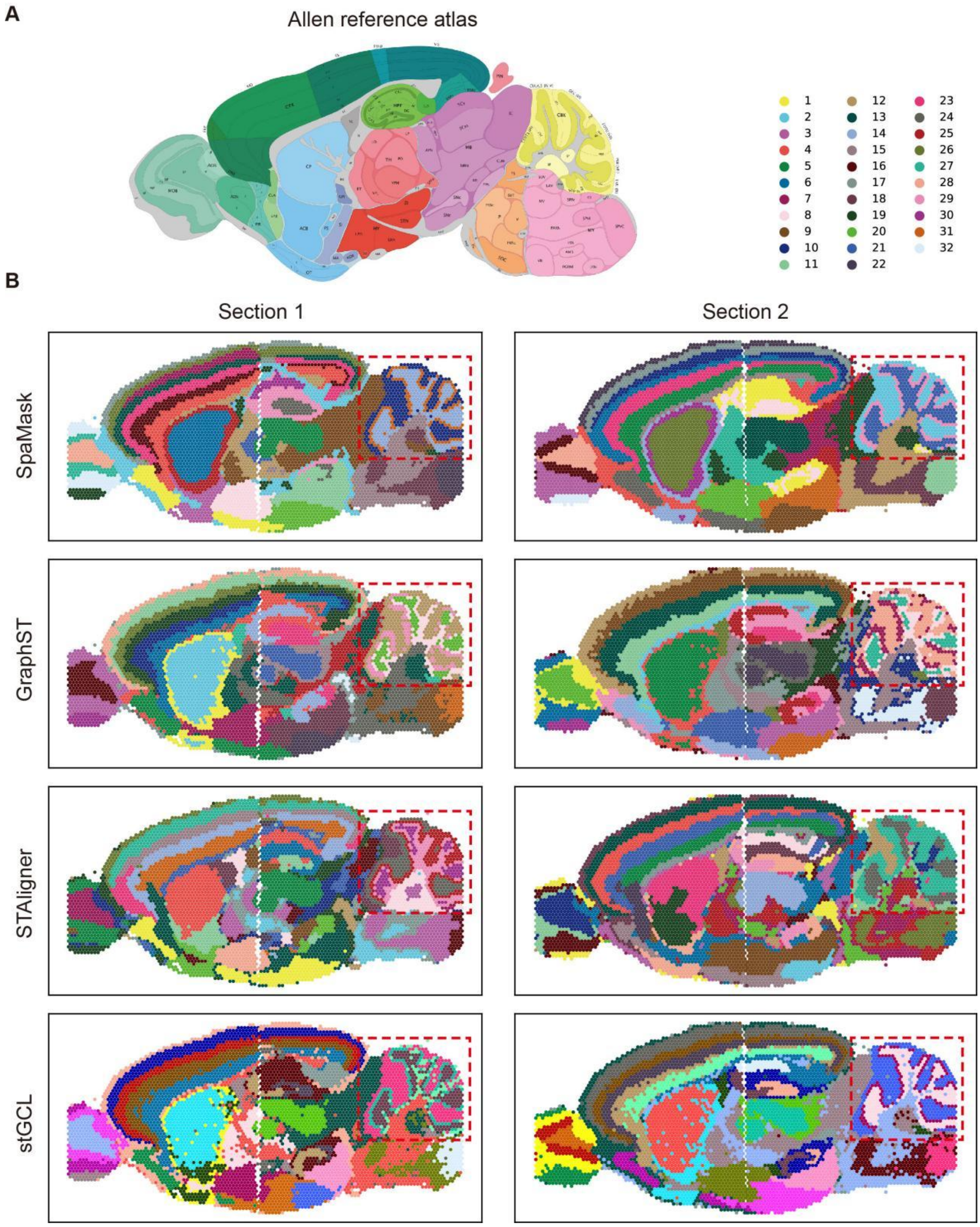

SpaMask achieves accurate horizontal integration across anterior and posterior datasets in mouse brains. (A) Annotated brain section image from the Allen Mouse Brain Atlas for reference. **(B)** Results of horizontal integration from two mouse brain samples, with each sample including slices from the anterior and posterior brain regions.
