# Supplementary material for: SpaMask: Dual masking graph autoencoder with contrastive learning for spatial transcriptomics
Source: PLoS Comput Biol. 2025 Apr 3;21(4):e1012881. doi: 10.1371/journal.pcbi.1012881 (PMC11968113; doi:10.1371/journal.pcbi.1012881)
Supplement: S14 Fig — (PDF) [file pcbi.1012881.s015.pdf]

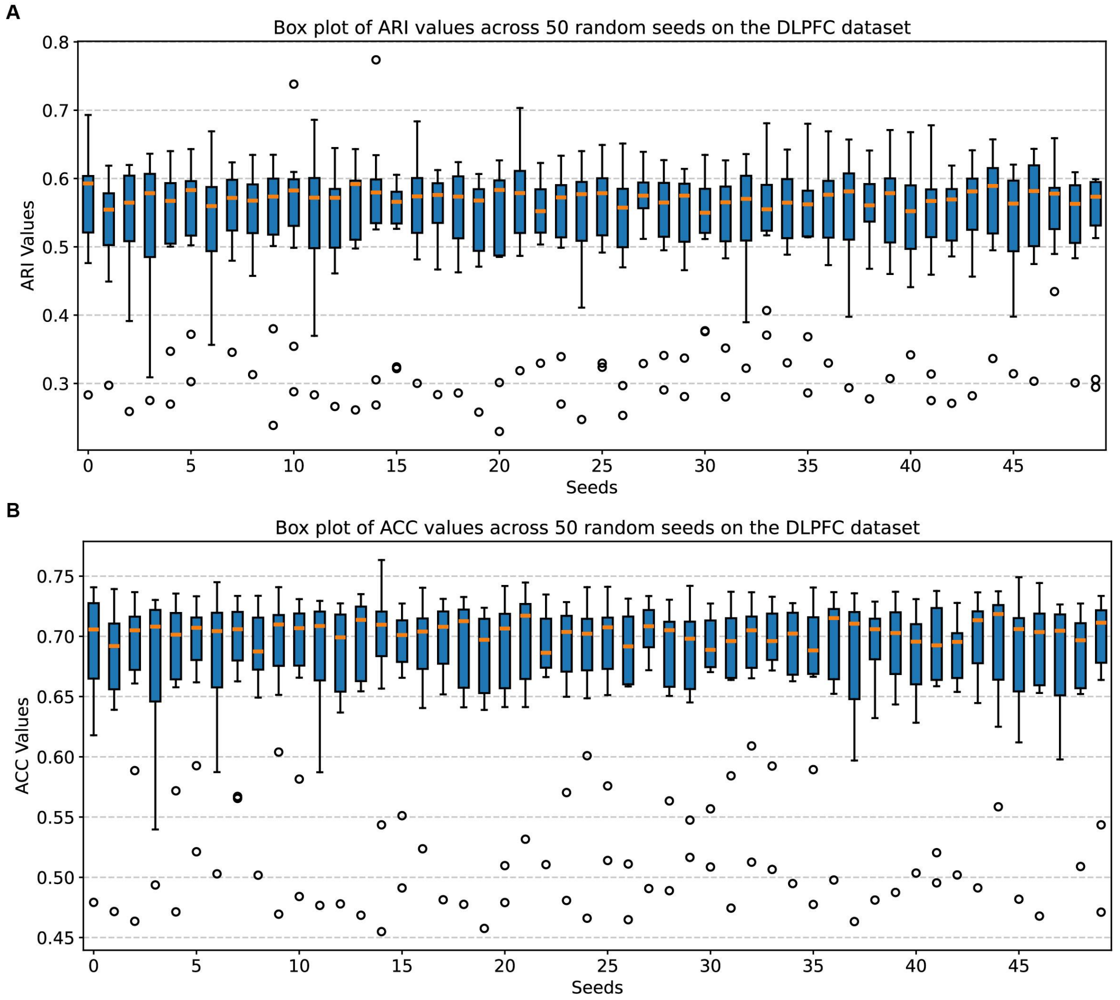

The clustering accuracy of SpaMask in all 12 sections under the default hyperparameters with different random seeds.
