# Supplementary material for: SpaMask: Dual masking graph autoencoder with contrastive learning for spatial transcriptomics
Source: PLoS Comput Biol. 2025 Apr 3;21(4):e1012881. doi: 10.1371/journal.pcbi.1012881 (PMC11968113; doi:10.1371/journal.pcbi.1012881)
Supplement: S1 Table — (PDF) [file pcbi.1012881.s016.pdf]

. **Summary of the datasets in this study.**

Platform	Tissue	Section	#Domains	#Spots	#Genes	Related figures
10x Visium	Human dorsolateral prefrontal cortex	151507	7	4,226	33,538	Figs 2, 3, S1, S2, S3, S4 and S5
		151508	7	4,384	33,538	
		151509	7	4,789	33,538	
		151510	7	4,634	33,538	
		151669	5	3,661	33,538	
		151670	5	3,498	33,538	
		151671	5	4,110	33,538	
		151672	5	4,015	33,538	
		151673	7	3,639	33,538	
		151674	7	3,673	33,538	
		151675	7	3,592	33,538	
		151676	7	3,460	33,538	
	Human breast cancer	\	20	3,798	36,601	Figs 4 and S6
	Mouse brain	Section1-Anterior		2,695	32,285	Fig S8
		Section1-Posterior		3,355	32,285	
		Section2-Anterior		2,825	32,285	
		Section2-Posterior		3,289	32,285	
ST	Human melanoma	\	4	293	16,148	Figs 4 and S7
osmFISH	Mouse somatosensory cortex	\	11	4,839	33	Figs 5 and S1
Stereo-seq	Mouse embryo	E9.5	12	5,913	23,015	Fig 5
	Mouse olfactory bulb	\	8	10,000	26,145	Fig 5
MERFISH	Mouse hypothalamic preoptic area	Bregma-0.04 mm	8	5,488	155	Figs 5 and S1
		Bregma-0.09 mm	8	5,557	155	
		Bregma-0.14 mm	8	5,926	155	
		Bregma-0.19 mm	8	5,803	155	
		Bregma-0.24 mm	8	5,543	155	
