# Supplementary material for: SpaMask: Dual masking graph autoencoder with contrastive learning for spatial transcriptomics
Source: PLoS Comput Biol. 2025 Apr 3;21(4):e1012881. doi: 10.1371/journal.pcbi.1012881 (PMC11968113; doi:10.1371/journal.pcbi.1012881)
Supplement: S2 Table — (PDF) [file pcbi.1012881.s017.pdf]

. **Summary of the clustering methods based on methodology, algorithm input and code link.**

Method	Methodology	Input Data			Link
		SRT data	Histology	scRNA-seq data	
SpaGCN (1)	GCNs	✓	✓	✗	https://github.com/jianhuupenn/SpaGCN
CCST (2)	Based-DGI GCL	✓	✗	✗	https://github.com/xiaoyeye/CCST
DeepST (3)	Variational graph autoencoder	✓	✓	✗	https://github.com/JiangBioLab/DeepST
SEDR (4)	Variational graph autoencoder	✓	✗	✗	https://github.com/JinmiaoChenLab/SEDR
STAGATE (5)	Graph attention autoencoder	✓	✗	✗	https://github.com/QIFEIDKN/STAGATE
GraphST (6)	GAE and based-DGI GCL	✓	✗	✗	https://github.com/JinmiaoChenLab/GraphST
DiffusionST (7)	Diffusion and ZINB	✓	✗	✗	https://github.com/cuiyaxuan/DiffusionST
STAligner (8)	STAGATE and MNN	✓	✗	✗	https://github.com/zhoux85/STAligner
Splane (9)	GCN and GAN	✓	✗	✓	https://github.com/QuKunLab/SPACEL
STitch3D (10)	ICP and GAT	✓	✗	✓	https://github.com/YangLabHKUST/STitch3D
SPIRAL (11)	GAN and GraphSAGE	✓	✗	✗	https://github.com/guott15/SPIRAL
stGCL (12)	GAT and ViT	✓	✓	✗	https://github.com/RuiGaolab/stGCL

References

1. Hu J, Li X, et al. SpaGCN: Integrating gene expression, spatial location and histology to identify spatial domains and spatially variable genes by graph convolutional network. Nature Methods. 2021;18(11):1342–1351.
2. Li J, Chen S, et al. Cell clustering for spatial transcriptomics data with graph neural networks. Nature Computational Science. 2022;2(6):399–408.
3. Xu C, Jin X, et al. DeepST: identifying spatial domains in spatial transcriptomics by deep learning. Nucleic Acids Research. 2022;50(22):e131–e131.
4. Xu H, Fu H, et al. Unsupervised spatially embedded deep representation of spatial transcriptomics. Genome Medicine. 2024;16(1):12.
5. Dong K, Zhang S, et al. Deciphering spatial domains from spatially resolved transcriptomics with an adaptive graph attention auto-encoder. Nature Communications. 2022;13(1):1739–1750.
6. Long Y, Ang KS, Li M, Chong KLK, Sethi R, Zhong C, et al. Spatially informed clustering, integration, and deconvolution of spatial transcriptomics with GraphST. Nature Communications. 2023;14(1):1155–1174.
7. Cui Y, Cui Y, Wang R, Nakai K, Ye X, Sakurai T, et al. DiffusionST: A diffusion model-based framework for enhancing spatial transcriptomics data quality and identifying spatial domains. Available at SSRN 4894131. 2024;.
8. Zhou X, Dong K, Zhang S. Integrating spatial transcriptomics data across different conditions, technologies and developmental stages. Nature Computational Science. 2023;3(10):894–906.
9. Xu H, Wang S, Fang M, Luo S, Chen C, Wan S, et al. SPACEL: deep learning-based characterization of spatial transcriptome architectures. Nature Communications. 2023;14(1):7603.
10. Wang G, Zhao J, Yan Y, Wang Y, Wu AR, Yang C. Construction of a 3D whole organism spatial atlas by joint modelling of multiple slices with deep neural networks. Nature Machine Intelligence. 2023;5(11):1200–1213.
11. Guo T, Yuan Z, Pan Y, Wang J, Chen F, Zhang MQ, et al. SPIRAL: integrating and aligning spatially resolved transcriptomics data across different experiments, conditions, and technologies. Genome Biology. 2023;24(1):241–267.
12. Yu N, Zhang D, Zhang W, Liu Z, Qiao X, Wang C, et al. stGCL: A versatile cross-modality fusion method based on multi-modal graph contrastive learning for spatial transcriptomics. bioRxiv. 2023; p. 2023–12.
