# Supplementary material for: SpaMask: Dual masking graph autoencoder with contrastive learning for spatial transcriptomics
Source: PLoS Comput Biol. 2025 Apr 3;21(4):e1012881. doi: 10.1371/journal.pcbi.1012881 (PMC11968113; doi:10.1371/journal.pcbi.1012881)
Supplement: S3 Table — (PDF) [file pcbi.1012881.s018.pdf]

. Mean (μ) and standard deviation (σ) of ARI and ACC metrics (expressed as $(\mu \pm \sigma) \times 100$) across four different platform datasets, evaluated using 50 independent experiments with random seeds for each method.

Dataset	SpaMask		GraphST		STAGATE		SEDR	
	ARI(%)	ACC(%)	ARI(%)	ACC(%)	ARI(%)	ACC(%)	ARI(%)	ACC(%)
151507	**60.79**$\pm$**3.66**	**72.00**$\pm$**2.01**	44.20$\pm$2.33	64.89$\pm$1.76	53.20$\pm$2.22	67.32$\pm$2.07	52.47$\pm$4.96	67.75$\pm$2.56
151508	**50.61**$\pm$**1.26**	**65.75**$\pm$**0.82**	49.27$\pm$2.03	64.40$\pm$1.35	47.53$\pm$4.07	62.81$\pm$2.34	49.96$\pm$2.01	64.81$\pm$0.70
151509	**59.73**$\pm$**0.83**	**71.46**$\pm$**0.85**	44.70$\pm$2.96	63.75$\pm$2.14	41.92$\pm$6.85	60.98$\pm$4.33	47.66$\pm$6.99	64.53$\pm$3.00
151510	**50.10**$\pm$**1.67**	**66.77**$\pm$**1.36**	49.33$\pm$3.90	64.13$\pm$1.73	49.54$\pm$4.33	63.34$\pm$2.38	47.89$\pm$3.47	63.58$\pm$1.86
151669	39.13$\pm$5.46	59.71$\pm$3.53	**51.93**$\pm$**4.17**	**62.91**$\pm$**2.25**	43.39$\pm$9.77	59.25$\pm$5.13	49.15$\pm$7.14	60.31$\pm$2.56
151670	30.84$\pm$6.60	49.51$\pm$2.91	**49.14**$\pm$**8.38**	**59.68**$\pm$**3.65**	37.51$\pm$4.41	56.81$\pm$0.89	35.88$\pm$1.92	52.65$\pm$1.74
151671	57.60$\pm$3.87	69.81$\pm$1.57	**63.00**$\pm$**0.90**	**73.62**$\pm$**0.79**	59.56$\pm$1.11	69.92$\pm$0.64	56.97$\pm$2.70	67.86$\pm$1.56
151672	**63.84**$\pm$**1.76**	**72.26**$\pm$**0.76**	63.52$\pm$1.90	71.38$\pm$0.70	59.53$\pm$5.00	68.66$\pm$1.44	56.29$\pm$1.69	67.30$\pm$1.98
151673	57.49$\pm$1.38	72.00$\pm$0.46	**63.64**$\pm$**0.80**	**73.52**$\pm$**0.37**	59.66$\pm$0.99	71.76$\pm$0.81	57.14$\pm$2.95	69.87$\pm$1.91
151674	**60.33**$\pm$**0.92**	**73.43**$\pm$**0.48**	50.94$\pm$6.98	65.13$\pm$4.07	54.41$\pm$8.83	66.25$\pm$8.05	56.35$\pm$5.33	68.77$\pm$3.70
151675	57.53$\pm$1.70	**71.12**$\pm$**1.16**	57.12$\pm$6.15	67.94$\pm$3.07	**59.60**$\pm$**1.66**	70.28$\pm$1.65	53.93$\pm$2.58	67.67$\pm$1.64
151676	**58.27**$\pm$**1.84**	**70.57**$\pm$**0.79**	57.02$\pm$1.76	66.34$\pm$1.64	49.06$\pm$8.51	61.97$\pm$6.58	52.44$\pm$4.48	66.29$\pm$2.68
Stereo	**71.37**$\pm$**1.16**	**73.19**$\pm$**0.92**	47.60$\pm$4.21	56.38$\pm$1.52	56.63$\pm$2.23	60.63$\pm$1.18	66.92$\pm$1.71	69.89$\pm$1.25
osmFISH	**60.22**$\pm$**4.44**	**69.69**$\pm$**2.19**	42.83$\pm$4.51	54.00$\pm$2.17	47.57$\pm$2.85	57.68$\pm$1.10	56.53$\pm$3.38	69.00$\pm$2.43
MERFISH	**47.38**$\pm$**3.48**	**59.09**$\pm$**2.20**	32.10$\pm$5.12	45.90$\pm$2.08	23.86$\pm$2.59	40.31$\pm$2.11	45.68$\pm$3.20	57.25$\pm$1.76
