# Supplementary material for: SpaMask: Dual masking graph autoencoder with contrastive learning for spatial transcriptomics
Source: PLoS Comput Biol. 2025 Apr 3;21(4):e1012881. doi: 10.1371/journal.pcbi.1012881 (PMC11968113; doi:10.1371/journal.pcbi.1012881)
Supplement: S4 Table — (PDF) [file pcbi.1012881.s019.pdf]

. Comparative analysis of computational resource consumption, including Model Runtime (MR/Seconds), GPU Memory Usage (GMU/MB), and Memory Caching (MC/MB), across various models.

Method	10x Visium			ST			osmFISH			Stereo-seq			MERFISH		
	MR	GMU	MC	MR	GMU	MC	MR	GMU	MC	MR	GMU	MC	MR	GMU	MC
SpaMask	63.15	**71.41**	2668	4.86	71.41	106	**5.34**	64.63	**222**	15.18	**71.41**	1050	24.77	70.89	1050
GraphST	77.8	2307	3034	3.66	84.07	116	5.64	158.21	258	14.52	913.08	1480	**5.67**	194.89	**310**
STAGATE	36.13	237.65	**1686**	**3.65**	67.85	106	5.57	**20.14**	504	19.99	403.98	1874	6.95	**30.02**	684
SEDR	**17.93**	140.37	2632	4.89	**66.18**	**74**	6.39	73.83	246	**9.69**	126.32	**762**	6.74	92.56	336
